# DRP1 Promotes BRAF^V600E^-Driven Tumor Progression and Metabolic Reprogramming in Colorectal Cancer

**DOI:** 10.3389/fonc.2020.592130

**Published:** 2021-03-02

**Authors:** Rayees Ahmad Padder, Zafar Iqbal Bhat, Zaki Ahmad, Neetu Singh, Mohammad Husain

**Affiliations:** ^1^ 409-Cancer Biology Laboratory, Department of Biotechnology, Jamia Millia Islamia, New Delhi, India; ^2^ Department of Zoology, PMB Gujrati Science College, Devi Ahilya Vishwavidyalaya, Indore, India; ^3^ Advanced Instrumentation Research Facility, Jawaharlal Nehru University, New Delhi, India

**Keywords:** BRAF^V600E^, DRP1, mitochondrial dynamics, metabolic reprogramming, colorectal cancer

## Abstract

**Background:**

Mitochondria are highly dynamic organelles which remain in a continuous state of fission/ fusion dynamics to meet the metabolic needs of a cell. However, this fission/fusion dynamism has been reported to be dysregulated in most cancers. Such enhanced mitochondrial fission is demonstrated to be positively regulated by some activating oncogenic mutations; such as those of KRAS (Kristen rat sarcoma viral oncogene homologue) or BRAF (B- rapidly accelerated fibrosarcoma), thereby increasing tumor progression/ chemotherapeutic resistance and metabolic deregulation. However, the underlying mechanism(s) are still not clear, thus highlighting the need to further explore possible mechanism(s) of intervention. We sought to investigate how BRAF^V600E^ driven CRC (colorectal cancer) progression is linked to mitochondrial fission/fusion dynamics and whether this window could be exploited to target CRC progression.

**Methods:**

Western blotting was employed to study the differences in expression levels of key proteins regulating mitochondrial dynamics, which was further confirmed by confocal microscopy imaging of mitochondria in endogenously expressing **BRAF^WT^** and **BRAF^V600E^** CRC cells. Proliferation assays, soft agar clonogenic assays, glucose uptake/lactate production, ATP/ NADPH measurement assays were employed to study the extent of carcinogenesis and metabolic reprograming in **BRAF^V600E^** CRC cells. Genetic knockdown (shRNA/ siRNA) and/or pharmacologic inhibition of Dynamin related protein1/Pyruvate dehydrogenase kinase1 (DRP1/PDK1) and/or BRAF^V600E^ were employed to study the involvement and possible mechanism of these proteins in **BRAF^V600E^** driven CRC. Statistical analyses were carried out using Graph Pad Prism v 5.0, data was analyzed by unpaired t-test and two-way ANOVA with appropriate post hoc tests.

**Results:**

Our results demonstrate that **BRAF^V600E^** CRC cells have higher protein levels of mitochondrial fission factor- **DRP1/pDRP1^S616^** leading to a more fragmented mitochondrial state compared to those harboring **BRAF^WT^**. This fragmented mitochondrial state was found to confer glycolytic phenotype, clonogenic potential and metastatic advantage to cells harboring **BRAF^V600E^**. Interestingly, such fragmented mitochondrial state seemed positively regulated by mitochondrial **PDK1** as observed through pharmacologic as well as genetic inhibition of **PDK1**.

**Conclusion:**

In conclusion, our data suggest that **BRAF^V600E^** driven colorectal cancers have fragmented mitochondria which confers glycolytic phenotype and growth advantage to these tumors, and such phenotype is dependent at least in part on **PDK1**- thus highlighting a potential therapeutic target.

## Introduction

Unlike other cellular organelles, mitochondria continuously change their morphology through balanced fission/fusion events- a phenomenon referred to as mitochondrial dynamics (1, 2) (for details refer to these reviews (1, 3–7) to meet the physiologic needs of a cell. This mitochondrial dynamics is chiefly regulated by small GTPases—DRP1 regulating fission while as optic atrophy factor 1 (OPA1) regulating inner membrane fusion, and mitofusin 1 & 2 (Mfn1 & 2) controlling outer membrane fusion (8). However dysregulation in such a balance may affect various cellular processes including programmed/autophagic cell death, proliferation and/or metabolic deregulation, which suggests that alteration in mitochondrial architecture may be the underlying cause behind many, if not all such transformative cell processes (4, 9, 10). Enhanced fission has been reported in various tumors (11–13), whose inhibition has significantly abrogated proliferation and induced apoptosis in lung or colon cancers (11, 12). Likewise mitochondrial fusion factor Mfn1 and/or Mfn2 have also been reported to show anticancerous role(s) in separate models of gastric and liver cancers (14, 15), and B cell lymphoma (16). Recently, though a Wnt inhibitor (ICG-001) was reported to inhibit DRP1 activity in a panel of colorectal cancer cell lines, and induce an early ER (endoplasmic reticulum) stress by activating ATF4, DDIT3, TRIB3 and ASNS, however clues about how oncogenic transformation implicates mitochondrial physiology and associated tumorigenesis of these cells are lacking (17). Contrary to these, mitochondrial fission has been reported to show anti-cancerous roles in separate models of rectal (18), lung, and colon cancers (19), making it more pertinent to study mitochondrial dynamics biology in CRC progression. Though cancer as a disease, is characterized by alterations directly linked to various pathways involving mitochondria (20), not much is mechanistically known about how oncogenic/tumor suppressor mutations directly affect mitochondrial physiology and associated tumorigenesis.

The RAS/RAF/MEK/ERK also known as Mitogen activated protein kinase (MAPK) pathway plays a central role in cellular homeostasis, survival, proliferation and apoptosis (21). Aberrant signaling or hyper activation of MAPK pathway has been shown in many tumors including melanomas, non-small cell lung cancer (NSCLC), pancreatic and colorectal cancers, chiefly due to mutations in RAS and BRAF proto-oncogenes (22). RAS and RAF mutations being the most prevalent oncogenic mutations in human cancers; BRAF mutations are reported in 7% of cancers but V600E alone accounts for more than 85% BRAF mutations in melanoma, about 50% in non-small cell lung carcinoma (NSCLC) and more than 95% in cholangiocarcinoma and hairy cell leukemia (23). It has been reported that nearly half of all colon cancers have constitutively active MAPK pathway owing to mutant RAS/BRAF with numbers still higher in bigger or advanced stage tumors. BRAF^V600E^ is the most prevalent mutation accounting for more than 90% in BRAF mutant colorectal cancers (23, 24). The V600E substitution has been reported to be associated with distinct clinico pathological features including proximal location, microsatellite instability, mucinous histology, higher age and advanced grade besides chemotherapeutic resistance (25, 26). Though the development of selective BRAF^V600E^ inhibitors- Vemurafenib (PLX4032) and dabrafenib (GSK2118436) for treating unresectable/ metastatic melanoma has met with a considerable success, the colorectal cancers harboring such mutation, however, have shown disappointing response rates while the underlying mechanisms remain elusive (26–28).

A characteristic feature of most, if not all cancerous cells is that they display a unique mode of nutrient utilization and hence metabolic phenotype compared to the normal tissues- a phenomenon known as metabolic reprograming, considered as one among the emerging hallmarks of cancer (29, 30). In line BRAF^V600E^ melanomas lead to a suppression of melanocyte master regulator- MITF and peroxisome proliferator Gama coactivator 1alpha- PGC 1α (a mitochondrial biogenesis factor) thereby decreasing oxidative phosphorylation (OXPHOS) (31–33). Further, reports about activated MAPK and enhanced mitochondrial fission have joined a growing list of evidence connecting oncogenic signaling with mitochondrial dynamics (34, 35). Additionally, dysregulated mitochondrial dynamics have been shown to affect normal mitochondrial function which in turn has physiological consequences on growing tumors (35, 36) thereby affecting cell proliferation (37, 38), metabolism (39) and apoptosis (40). Such cancer hallmark capabilities reflect, at least, in part some acquired aberrations in mitochondrial function- preferably a shift from OXPHOS to glycolysis (41); the latter is achieved through inhibition of pyruvate dehydrogenase complex (PDH) by PDK1 thus inhibiting/abrogating the entry of Acetyl CoA (ac CoA) to fuel tricarboxylic acid (TCA) cycle and OXPHOS (42, 43). Despite, a similar role of both DRP1 and PDK1 in promoting certain cancer phenotypes and fueling Warburg effect, the physiological crosstalk between these proteins is completely unknown.

In this context, we sought to investigate the role of DRP1 in some colorectal cancerous cell models driven by endogenous BRAF^V600E^. We find that DRP1 is indispensable for BRAF^V600E^ driven cancer progression in CRC cells. Mechanistically, DRP1 is either overexpressed and/or activated at P^S616^ giving rise to a more fragmented mitochondrial phenotype in BRAF^V600E^ CRC cells compared to BRAF^WT^. Such a fragmented mitochondrial phenotype is essential to enhanced Warburg effect, cellular proliferation and migration/invasion as observed through DRP1 silencing either by pharmacologic or genetic means. Interestingly, this BRAF^V600E^ driven CRC progression was found to be regulated via mitochondrial PDK1 as observed by pharmacologic/genetic knockdown approaches. Overall these results highlight a *hitherto* unknown function of glycolytic gatekeeper PDK1 in regulating DRP1 mediated mitochondrial fission in BRAF^V600E^ CRC. Together, our data identify that mitochondrial fission could be a therapeutic vulnerability of BRAF^V600E^ CRC, and targeting such phenotype would pave ways for better therapeutic intervention in such patients.

## Materials and Methods

### Cell Lines

The colorectal cancer cell lines, Colo-205 and HT-29 harboring BRAF^V600E^, and SW48 with a BRAF^WT^ (44) in endogenous setting/s were procured from National Centre for Cell Sciences, Pune India (NCCS, Pune). All the cell lines were maintained in Dulbecco’s Modified Eagles Medium (DMEM, Gibco) with 2-mM glutamine, 1X penicillin/streptomycin (Gibco) and 10% Fetal Bovine Serum (FBS, Gibco) at 37°C and 5% CO_2_ under humidified conditions in a cell culture incubator (Shell labs).

### Cell Proliferation Assay

The cells growing in log phase (approx. 70% confluence) were trypsinized and seeded in 12 well plates at a density of 1.0 x 10^4^ -1.5 x 10^4^ cells per well in quadruplet in four such replicates (1–4). Every day, duplicate wells from each experimental group were trypsinized and manually counted (total cell number) using a Neubauer chamber to generate a cell growth curve over a period of 4 days. For drug treated groups, the cells were treated first either with small molecule mdivi-1 (20 µM) or vemurafenib (15 µM) for 48 h and then the treated cells were trypsinized and seeded for assessing proliferation over time (1–4 days). For PDK1 inhibition, siRNA transfected cells along with corresponding mocks (sc-siRNA groups) were seeded after 48 h post transfection and the proliferation assessed as above. Cell proliferation in stably expressing shDRP1 and sc-shRNA cells was also evaluated as above.

### Anchorage Independent Growth in Soft Agar

The assay was performed as described previously with slight modifications (45). Briefly, the uniform cell suspensions (in 0.35% noble agar in DMEM; 1:1 ratio) were poured onto high percentage pre solidified noble agar medium (in 0.70% noble agar in DMEM; 1:1 ratio) into 12 well plates at a density of 2.5 x 10^3^ cells per well in triplicate. The wells were covered with complete media to avoid desiccation of agar and the plates incubated for 3–4 weeks, with occasional replacement of fresh media every 4–5 days. Post incubation period the top media was removed and the agar stained using 0.01% of crystal violet dye for 15 min at room temperature so as to ensure complete uniform staining of the agar wells. After staining, the wells were washed gently with distilled water several times so as to de-stain the background; the plates were scanned on white background using Chemidoc XRS^+^ from Bio Rad to count the visible cell colonies in different groups and calculate the plating efficiency. For drug treated groups, the cells were seeded along with low dose of dichloro acetate (DCA, 10 mM) or vemurafenib (5 µM).

### Stable Cell Generation and siRNA Transfection

For stable knock down of mitochondrial fission GTPase- DRP1, Colo-205, and HT-29 cells were transfected with shDRP1 plasmid (pSuper-Retro-Puro-Drp1-shRNA), a gift from David Kashatus (Addgene plasmid # 99385; http://n2t.net/addgene:99385; RRID:Addgene_99385) or sc-shRNA plasmid (pSIH1-puro-control shRNA), a gift from Frank Sinicrope (Addgene plasmid # 26597; http://n2t.net/addgene:26597; RRID:Addgene_26597) (34, 46), using Lipofectamine 3000 (Thermo scientific) as per manufacturer instructions. Briefly, low passage (usually 5-10) healthy cells were seeded at high density into 35 mm dishes and after 24 h, transfected with either shDRP1 or sc-shRNA plasmid/s. After 10 h of transfection, cells were replenished with fresh media until 48 h at which time the cells were selected using 0.30 (Colo-205) or 0.35 (HT-29) µg/ml puromycin in 10 cm dishes for 7–10 days. Gene silencing was confirmed by western blot, backup stocks cryopreserved and the cells were used for further experimentation as discussed.

Oligonucleotide transfections were given transiently for siRNA control (sc-siRNA), sense- UUCUCCGAACGUGUCACGUTT anti sense-ACGUGACACGUUCGGAGAATT and siRNA PDK1, sense- GGAUGCUAAAGCUAUUUAUTT anti sense AUAAAUAGCUUUAGCAUCCTT oligos (IDT), sequences described previously (47), using Lipofectamine 3000 reagent (Thermo scientific) as per manufacturer instructions. Briefly the cells at 60-70% confluence were serum starved for 3–4 h prior to transfection, after which the cells were transfected with 40 nM of indicated Oligos. Transfection media was changed after 8 h post transfection and transfection efficiency assessed after 72 h post transfection using western blot. For *in vitro* proliferation, metabolism or metastasis related assays the siRNA transfected cells were trypsinized at 48 h (post transfection) and seeded at appropriate densities into 12 well plates or 35 mm dishes as appropriate.

### Wound Healing and Transwell Cellular Invasion Assays

Wound healing assay was carried out for HT-29 cells and not for Colo-205 (owing to their semi adherent nature) for assessing cell migration. Briefly 1 x 10^5^ cells were seeded into respective wells of a 24 well plates in triplicates and allowed to reach a confluency of approximately >90%. Then a scratch was made (after serum starvation for 6–8 h) using a 10 µl pipette tip along the diameter of each well to create a wound like structure. This was followed by gently aspiring the media and washing the wells briefly twice using 1x PBS to completely remove the detached cells. Then the wells were replenished with either 0.5% FBS containing media or 0.5% FBS containing either 15 mM 2-DG (deoxyglucose) or 20µM mdivi-1 as indicated. Pictures were taken at 0, 12 and 24 h post scratching and the migratory potential determined by calculating the wound closure in respective groups using Image J software.

The invasion assays were carried out using transwell inserts for both HT-29 as well as Colo-205 cell lines. Briefly 1 x 10^5^ cells were seeded in serum free medium (200 µl volume) or 15 mM 2-DG onto 24 well plate transwell inserts (SPL Insert, cat no. 36224), coated with 40 µl ECM. The lower chamber of the well was loaded with 600–700 µl of 10% FBS media which served as a chemo attractant to stimulate cell invasion across transwell pores. The cells were pretreated with 10 µM vemurafenib for 24 h before carrying invasion assays. Similarly, either stably expressing shDRP1 or pre-transfected (48 h prior) Si-PDK1 groups were processed as above. After 24 h, the respective inserts were washed gently in 1x PBS, fixed in pre-chilled 70% ethanol for 15 min and stained using 0.1% crystal violet dye for 10–15 min. Then the inserts were swabbed from inside using moist cotton swabs to remove the cells or the matrigel, followed by de-staining the extra dye in water. Pictures of the invaded cells were taken at random fields using an inverted microscope to calculate invasion of cells in different groups.

### Western Immunoblotting

The cells (as indicated) were trypsinized and lysed in Pierce IP lysis buffer (Thermo Fisher Scientific) supplemented with 0.5% sodium deoxycholate, 0.1% SDS along with 1x Phos stop and Sigma fast (Sigma Aldrich) for 10–15 min on ice. The lysate was spun at 10000 rpm for 10 min at 4°C; supernatant taken and total protein was quantified using BCA kit (Thermo Scientific). Equal amounts of protein (50-80 µg) were resolved on SDS-PAGE gels, followed by transfer of the resolved proteins onto PVDF membrane (Bio Rad) using wet transfer system at 75 V for 1.5 h. The membranes were then blocked with 5% nonfat milk in TBST for 40 min and probing overnight with indicated primary antibodies at 4°C. Next day, the primary probed membranes were washed thrice for 5 min each in 1x TBST and probed with appropriate HRP conjugated secondary antibodies at room temperature for 1 h. The antigen signals were captured using chemiluminescence based detection, the signals were captured onto x-ray film (Amersham). The primary antibodies used were against DRP1 (SC-271583, Santa Cruz), pDRP1^S616^ (PA5-64821, Thermo Scientific), Mfn1/2 (NBP1-51841/STJ-94105, R & D/St Johns Laboratory), β- actin (NB600-501, Novus Biologicals), E cadherin (NBP2-19051, Novus Biologicals), N cadherin (NBP1-48309, Novus Biologicals), Vimentin (STJ140133, St Johns Laboratory), PDK1 (STJ24945, St Johns Laboratory), p-MEK 1/2 (sc-81503), MEK 1/2 (sc-81504) and the corresponding secondary antibodies used were either goat anti-rabbit IgG-HRP (NB7160, Novus Biologicals) or goat anti-mouse IgG-HRP (NB7539, Novus Biologicals).

### Immunofluorescence and Mitochondrial Morphology Analysis

Immunofluorescence was carried out as previously described with some modifications (34, 48). Briefly, cells were seeded at low density on glass coverslips in 12 well plates and where ever indicated, treated with appropriate drug/s (for 24 h) the next day post seeding. Then live cells were stained with 100 nM Mito Tracker CMX Ros (Thermo Scientific, cat M7512) in the same media for 30–40 min. After washing with 1x PBS several times the cells were fixed with pre chilled absolute methanol at -20°C for 15 min, thereafter the coverslips were rinsed with 1x PBS and stained with 25 nM DAPI in PBS for 5 min. Finally the coverslips were washed a couple of times using 1x PBS for 5 min each and subsequently mounted using 0.5 N-propyl gallate mounting solution (in 80% glycerol/20mM Tris pH 8.0). The mounted coverslips were sealed with nail paint and stored at 4°C in dark until imaged using a confocal imaging system from Olympus (Olympus FluoView FV-1000). A cell with more than 50% fragmented mitochondria was designated as fragmented, while a cell with 70-80% elongated mitochondria was designated as tubular/elongated and a cell with around 50% short rod like mitochondria was designated with intermediate phenotype (49). About 30-40 cells were quantified per group using Image J V 1.53c (NIH), equipped with mitochondrial network imaging (MINA) macro.

For immunocytochemistry the cells were seeded and treated (where appropriate) as above, the media was removed and the cells were rinsed a couple of times with 1x PBS and subsequently fixed as above followed by permeabilizing with 0.2% triton X-100 (in 1x PBS) for 10 min at room temperature. Then the cells were rinsed with 1x PBS and blocked in blocking buffer for half an hour at room temperature, followed by probing the cover slips with indicated primary antibodies (PDK1 MA5-15797, & pDRP1^S616^ PA5-64821, Thermo Scientific) at appropriate dilutions for 2 h at room temperature. Primary probed coverslips were washed thrice in 1x PBS for 5 min each before being secondary probed with appropriate secondary antibodies (Alexa fluor 488-A27034 & Alexa fluor 647-A21236, Thermo Scientific) at appropriate concentrations for 1 h at room temperature in dark. Afterwards, the coverslips were washed thrice in 1x PBS for 5 min each followed by DAPI staining and mounting as above.

### Measurement of Glucose Uptake/Lactate Production, ATP, and NADPH

Glucose uptake and lactate production was measured in the spent media using respective kits (Eton Bioscience, SKU- 1200032002 & 1200012002) as per manufacturer instructions. Similarly ATP levels were measured using ATP Colorimetric/Fluorometric Kit (Bio vision, K354-100) and NADPH levels were quantified using Amplite Colorimetric NADPH Assay Kit (AAT Bioquest, 15272) as per manufacturer instructions.

### Statistical Analysis

All statistical analyses were carried out using Graph Pad Prism v 5.0. Data acquisition or analysis was not blinded. Differences between groups were analyzed either by unpaired t-test or two-way ANOVA as applicable with appropriate post hoc tests as indicated. All the data are represented as mean± SEM of at least three replicates unless otherwise stated. *P* values corresponding to P≤0.05 were considered statistically significant.

## Results

### BRAF^V600E^ Colorectal Cancer Cells Tend to Have More Fragmented Mitochondria

**Figure 1:**
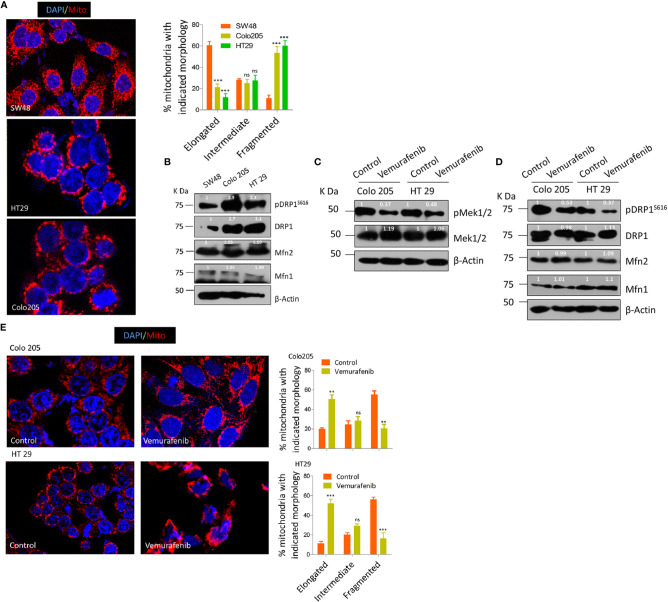
BRAF^V600E^ CRC cells have more fragmented mitochondria. **(A)** Representative confocal micrographs (2.5x zoom; 60x original magnification) of BRAF^WT^ and BRAF^V600E^ CRC cells, stained using mitotracker CMX Ros (mitochondria; red) and DAPI dyes (nuclei; blue), morphology quantified from 30 to 40 cells (n=3, mean± SEM), statistical analysis was done by two way ANOVA, followed by Bonferroni post-tests comparing replicate means by column with corresponding p values shown. **(B)** Western blot showing higher expression levels of fission mediator DRP1 as well its activating phosphorylation pDRP1^S616^, with no visible variation in fusion mediator MFN 1&2 in such cells. **(C)** Western blot showing vemurafenib inhibits MEK 1/2, a downstream effector of BRAF^V600E^. **(D)** Western blot showing vemurafenib treatment in BRAF^V600E^ CRC cells inhibits fission mediator DRP1 with no significant effect on fusion proteins (Mfn1 & 2). **(E)** Representative confocal micrographs (2.5x zoom; 60x original magnification) of vemurafenib treated BRAF^V600E^ CRC cells, stained and quantified as in A.

Previous studies demonstrating a constitutive fragmented mitochondrial morphology in oncogenic KRAS or BRAF driven cancers (34, 48, 50, 51) prompted us to seek whether such endogenously activating mutations in colorectal cancerous cell lines have any role in tumorigenesis. To this end, we evaluated as to what extent endogenous BRAF^V600E^ CRC cells lead to a fragmented mitochondrial phenotype compared to BRAF^WT^. It was found that HT29 and Colo205 cells (from here onwards BRAF^V600E^) had a more fragmented mitochondrial phenotype compared to SW48 (from here onwards BRAF^WT^) CRC cells (**Figure 1A**). Since mitochondrial morphologies are regulated by various fission/fusion dynamins, mainly dynamin related protein 1 (DRP1)/mitofusins 1 & 2 (Mfn1 & 2) respectively, our next interest was to investigate whether such variation in mitochondrial morphologies was related to pro-fission or fusion mediators alone or both. It was found that BRAF^V600E^ cells had higher levels of pro-fission mediator DRP1 as well as its activated form pDRP1^S616^ compared to BRAF^WT^ cells; however, the fusion mediating protein expression (Mfn1/2) did not vary significantly (**Figure 1B**), indicating the more prominent role of pro-fission GTPase DRP1 in BRAF driven CRC tumors.

To rule out whether such mitochondrial phenotype is the characteristic of BRAF^V600E^ in these cells with minimal possibility of involvement of other oncogenic events, we blocked the constitutive activation of MEK/ERK pathway downstream of BRAF^V600E^ in these cells. The selective BRAF^V600E^ inhibitor (vemurafenib, 10 µM for 24 h) significantly abrogated the constitutive activation of MEK/ERK pathway as observed through the reduction in p-MEK 1/2, a downstream effector of BRAF and an activator of ERK (**Figure 1C**). Interestingly, such inhibition of BRAF^V600E^ also led to a reduction in mitochondrial fission as observed through a reduction in pDRP1^S616^ levels (**Figures 1D, E**). These data provide evidence that mitochondria in BRAF^V600E^ CRC cells are more fragmented and such a mitochondrial phenotype is driven by BRAF^V600E^ mutation.

### Higher Mitochondrial Fission Rates Confer Growth Advantage to BRAF^V600E^ Colorectal Cancer Cells

**Figure 2:**
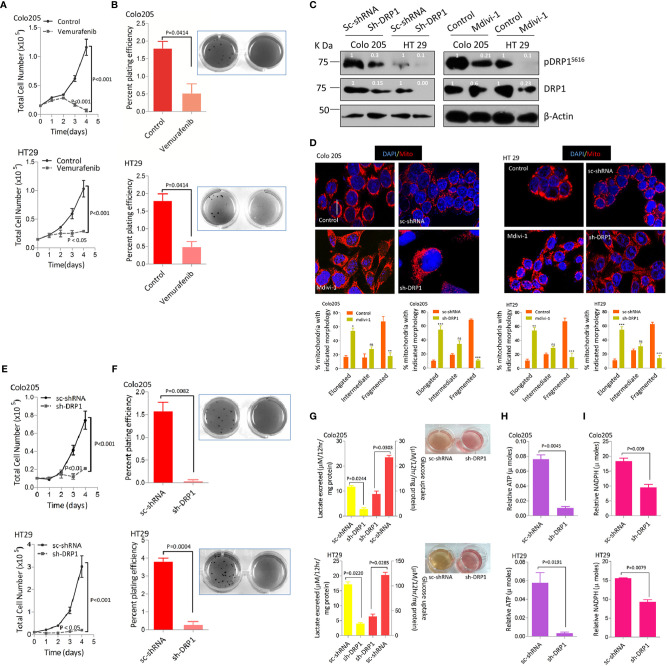
More fragmented mitochondrial phenotype leads to a more tumorigenic potential and reprogramming in glucose metabolism in BRAF^V600E^ CRC cells. **(A)** Inhibition of BRAF^V600E^ using vemurafenib leads to reduction in cell proliferation in BRAF^V600E^ cells (n=4, mean± SEM), statistical analysis was done by 2 way ANOVA, followed by Bonferroni post-tests comparing replicate means by row with corresponding p values shown. **(B)** Inhibition of BRAF^V600E^ using vemurafenib leads to reduction in clonogenic growth in BRAF^V600E^ cells (n=3, mean± SEM), statistical analysis was done by unpaired t-test followed by Welch’s correction assuming unequal variance in means, corresponding p values shown. **(C, D)** Western blots showing silencing of DRP1 in BRAF^V600E^ CRC cells either by mdivi-1 or using stable gene silencing by shRNA leads to reduction in mitochondrial morphology as shown in confocal micrographs (2.5x zoom, 60x original magnification), morphology quantified in 30–40 cells (n=3, mean± SEM), statistical analysis was done by two way ANOVA, followed by Bonferroni post-tests comparing replicate means by column with corresponding p values shown. **(E, F)** Stable knockdown of DRP1 in BRAF^V600E^ CRC cells leads to a reduction in cell proliferation (n= 4, mean± SEM) as well as clonogenic rates in soft agar (n=3, mean± SEM); statistical analysis for proliferation was done by 2-way ANOVA followed by Bonferroni post-tests comparing replicate means by row with corresponding p values shown, and statistical analysis for soft agar clonogenic assay was done by unpaired t-test followed by Welch’s correction assuming unequal variance in means, corresponding p values shown. **(G)** Lactate production and Glucose consumption rates post DRP1 silencing in BRAFV600E CRC cells (n=3, mean± SEM); soft agar wells of indicated cells showing a reduction in yellowing of soft agar and hence glucose fermentation rate, **(H, I)** Relative ATP and NADPH levels post DRP1 silencing in BRAF^V600E^ CRC cells (n=3, mean± SEM), statistical analysis was done by unpaired t-test followed by Welch’s correction assuming unequal variance in means, corresponding p values shown.

Mitochondrial fission driven by activating phosphorylation of DRP1 (pDRP1^S616^) downstream of both KRAS or BRAF pathway/s confers a growth advantage as well as metabolic shift towards a more glycolytic phenotype in various models of cancer (34, 50). We sought to perceive the *hitherto* unknown approach of whether mitochondrial fission could be utilized as a therapeutic modality in BRAF^V600E^ CRC. To answer this question the tumorigenic propensity between BRAF^V600E^ and BRAF^WT^ CRC cells was explored by evaluating cell proliferation ability at varying time periods (1–4 days) and anchorage independent growth using soft agar assay. It was found that BRAF^V600E^ CRC cells had significantly higher cell growth rates compared to BRAF^WT^ (**Supplementary Figures 1A, B**). To rule out if such tumorigenic propensity is regulated by BRAF^V600E^ signaling upstream of DRP1 (mitochondrial fission), we treated Colo 205 and HT 29 cells with PLX4032 (vemurafenib), a selective BRAF^V600E^ inhibitor. It was found that blocking BRAF not only abrogated cell proliferation but also growth in soft agar (**Figures 2A, B**), indicative of an upstream role of BRAF^V600E^ over DRP1 (mitochondrial fission). To further decipher whether such tumorigenic potential correlates to higher fragmented mitochondrial state, a pharmacologic (mdivi-1), or genetic knockdown of fission mediating protein-DRP1 using shDRP1 (generating stably expressing shDRP1) was carried out in Colo 205 and HT 29 cells. It was found that both pharmacologic and genetic knockdown of DRP1 reduced mitochondrial fission (**Figures 2C, D**) as well as cell proliferation (**Figure 2E**) compared to respective mock groups. In addition, anchorage independent growth of shDRP1 cells was also abrogated to significant levels compared to non-silenced mocks (**Figure 2F**).

A surprising observation during anchorage independent growth assay was the more yellowing of soft agar by HT29 and Colo205 cells compared to SW48 cells, indicating a drop in pH and hence an overutilization of glucose and its subsequent conversion to lactate (**Supplementary Figure 1C**). This prompted us to measure glucose consumption and/or lactate production/excretion rates in these cells. It was observed that excessive yellowing of soft agar was positively linked to overutilization of glucose and production of lactate by these cells (**Supplementary Figure 1C**). To establish whether such dichotomy in glucose metabolism is characteristic of BRAF^V600E^, we inhibited BRAF using its inhibitor vemurafenib. We observed the reversal of yellowing of soft agar, in line with a reduction in glucose fermenting propensity post BRAF inhibition (**Supplementary Figure 1D**). We also studied the effect of BRAF inhibition on cellular ATP and NADPH levels owing to their importance in generating surplus energy and biomass, besides maintaining the redox state of proliferating cells (52, 53). It was observed that blocking BRAF^V600E^ significantly reduced ATP production as well as NADPH generation (**Supplementary Figures 1E, F**). These data indicate that BRAF^V600E^ CRC cells are metabolically distinct in fermenting glucose at a faster rate compared to BRAF^WT^ cells, which possibly could be indispensable to their mitochondrial morphology and hence enhanced tumorigenicity. To further ascertain whether such shift in metabolism correlates to mitochondrial fission, we knocked down the expression of fission mediator DRP1 by genetic means generating stably expressing shDRP1 cell clones. Interestingly, the yellowing of soft agar was completely restored in shDRP1 groups compared to respective mocks (**Figure 2G**), indicating a decline in glucose fermentation rate after DRP1 silencing in otherwise glucose fermenting BRAF^V600E^ cells. In this context, the glucose fermentation rate post DRP1 silencing was evaluated in these cells and it revealed that stably expressing shDRP1 BRAF^V600E^ cells exhibit significantly reduced rate(s) of glucose uptake and/or lactate production (**Figure 2G**). Moreover, DRP1 silencing also led to a significant reduction in ATP and NADPH levels (**Figures 2H, I**), indicating a decline in reducing power generation as well as energy production, otherwise necessary to maintain enhanced proliferation and redox state in cancerous cells.

### Mitochondrial Pyruvate Dehydrogenase Kinase 1 Regulates Dynamin-Related Protein 1 Mediated Mitochondrial Fission in BRAF^V600E^ Colorectal Cancer Cells

**Figure 3:**
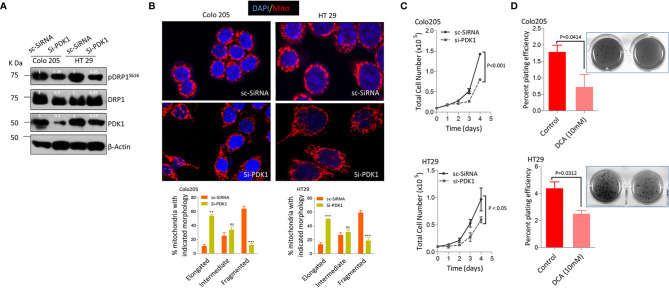
Pyruvate dehydrogenase kinase 1 (PDK 1) regulates DRP1 mediated mitochondrial fission and glycolytic phenotype in BRAF^V600E^ cells. **(A, B)** Western blot and representative confocal micrographs (2.5x zoom, 60x original magnification) of si-PDK1 silenced BRAF^V600E^ CRC cells, showing a reduction in both pDRP1^S616^ levels as well as mitochondrial fission after PDK1 knockdown, 30-40 cells were quantified (n=3, mean± SEM), red= mitochondria, CMX Ros stained and blue= Nuclei, DAPI stained, statistical analysis was done by two way ANOVA, followed by Bonferroni post-tests comparing replicate means by column with corresponding p values shown. **(C, D)** Genetic and pharmacologic knockdown of PDK1 in BRAF^V600E^ CRC cells leads to a reduction in cell proliferation (n= 4, mean± SEM) and clonogenic rates in soft agar (n=3, mean± SEM) respectively; statistical analysis for proliferation was done by 2-way ANOVA followed by Bonferroni post-tests comparing replicate means by row with corresponding p values shown, and statistical analysis for soft agar clonogenic assay was done by unpaired t-test followed by Welch’s correction assuming unequal variance in means, corresponding p values shown.

The dependence of various cancers on RAS/RAF/MAPK pathway to rewire their glucose metabolism and the importance of mitochondrial PDK1 as a central regulatory switch at the crossroads of glycolysis to oxidative phosphorylation (OXPHOS) (47, 54), besides higher glucose fermentation rate/s shown by BRAF^V600E^ cells prompted us to investigate if PDK1 had any role in such metabolic reprograming and cancer progression. Therefore, siRNA mediated silencing of PDK1 was undertaken, which was found to reduce pDRP^S616^ levels with concomitant reduction in glucose fermentation rate (**Figure 3A**, & **Supplementary Figures 2A, B**). The PDK1 silencing, additionally, led to a more elongated mitochondrial phenotype compared to control group (**Figure 3B**), indicative of a role of PDK1 in regulating mitochondrial fission in BRAF^V600E^ CRC cells. Next we sought to find whether such ablation of PDK1 had any role in tumorigenicity of these cells, which was found to reduce cell growth *in-vitro* (**Figure 3C**). The pharmacological inhibition of PDK1 by dichloro acetate (DCA, at lower dose of 10mM) reduced the anchorage independent growth in soft agar (**Figure 3D**). Further, siRNA mediated silencing of PDK1 led to a significant reduction in both ATP as well as NADPH levels (**Supplementary Figures 2C, D**). These data demonstrate that mitochondrial fission regulates the carcinogenic potential as well as glycolytic metabolism of BRAF^V600E^ CRC cells and such phenotype is dependent in part on the glycolytic gatekeeper- mitochondrial PDK1.

Additionally we tried to observe the effect(s) of silencing either PDK1 or DRP1 in BRAF^V600E^ (Colo205 & HT29) cells to ascertain whether mitochondrial elongation was due to an increase in fusion proteins too, however, the protein levels of both Mfn1 & 2 remained unchanged (**Supplementary Figure 2E**). This indicates that mitochondrial fission in these cells is regulated primarily by DRP1/pDRP1^S616^ activity and that silencing either PDK1 or DRP1 has no effects on fusion mediating proteins.

### Mitochondrial Fission Mediator Dynamin-Related Protein 1 Regulates Cellular Migration and Invasion *via* Glucose Metabolic Reprograming Episode(s)

**Figure 4:**
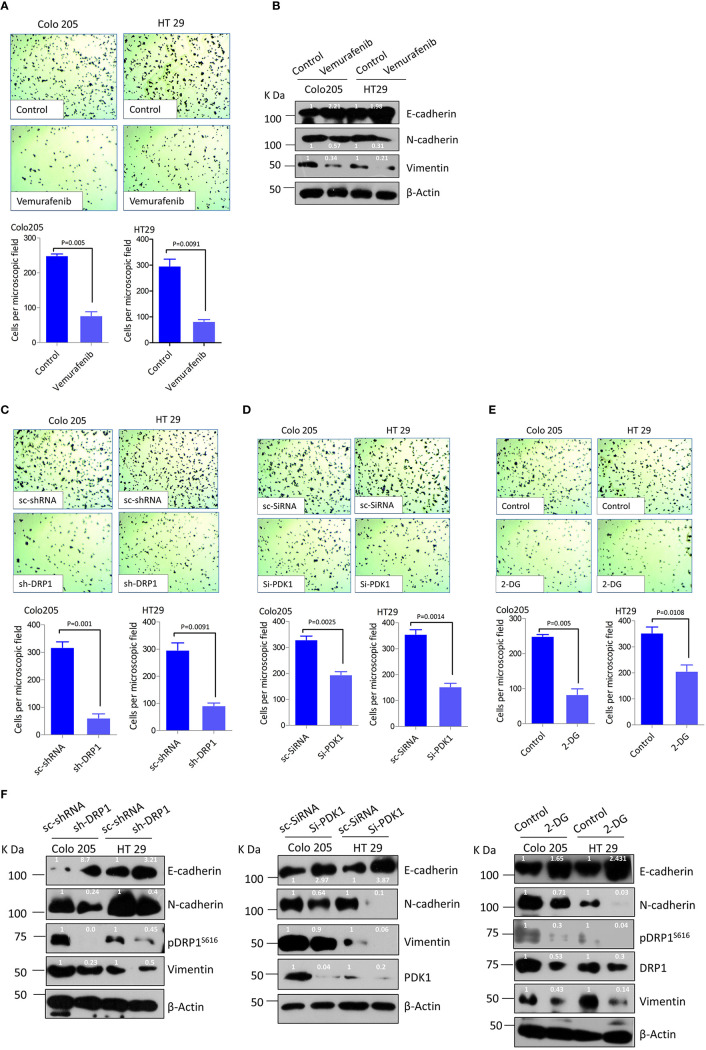
Mitochondrial fission regulates migration and invasion in BRAF^V600E^ CRC cells through glucose metabolic reprograming. **(A)** Relative cell invasion post vemurafenib treatment in BRAF^V600E^ cells (n=3, mean ± SEM); statistical analysis was done by unpaired t-test followed by Welch’s correction assuming unequal variance in means, corresponding p values shown. **(B)** Western blot showing a reduction in EMT markers upon vemurafenib treatment in BRAF^V600E^ cells. Relative cellular invasion post **(C)** DRP1 silencing, **(D)** PDK1 silencing, and **(E)** 2-deoxyglucose treatment in Colo205 and HT29 cells (n=3, mean± SEM); statistical analysis was done by unpaired t-test followed by Welch’s correction assuming unequal variance in means, corresponding p values shown. **(F)** Western blots showing a reduction in epithelial mesenchymal transition (EMT) markers upon DRP1 silencing, PDK1 knockdown, and 2-DG treatment in Colo205 and HT29 cells.

Since BRAF^V600E^ confers metastatic predisposition to CRC (55–57), and a pro-metastatic role of mitochondrial fission in other cancers (58–60), we sought to investigate if mitochondrial fission has any therapeutic relevance towards metastatic predisposition of BRAF^V600E^ CRC. First, we treated BRAF^V600E^ CRC cells with vemurafenib to see if blocking BRAF activity hampers their invasive potential. We found a significant reduction in cell invasion through matrigel coated transwell inserts in drug treated cells (**Figure 4A**). At molecular level vemurafenib induced E-cadherin expression while reducing N-cadherin and Vimentin levels (**Figure 4B**). These data suggest that constitutive activation of MEK/ERK pathway downstream of BRAF^V600E^ in CRC cells is responsible for a more invasive and/or mesenchymal propensity of these cells.

Next, we sought to find if such metastatic propensity is attributed to mitochondrial dynamics and the underlying metabolic relevance. It was observed that both pharmacologic and genetic knockdown of DRP1 significantly reduced wound healing capacity in HT29 cells (**Supplementary Figures 3A, B**). Further, to ascertain whether such migration was linked to glucose metabolic reprograming, 2-DG (2-deoxyglucose), a competitive inhibitor of glucose import into the cancerous cells, was used to hamper their bioenergetic needs (by reducing glycolysis). Interestingly, wound healing capacity was abrogated significantly in 2-DG treated cells (**Supplementary Figure 3B**), indicating a dependence of such cellular migration on glucose reprogramming. Similarly, PDK1 silencing also abrogated such wound healing capacity, though to a lesser extent (**Supplementary Figure 3C**), further indicating glycolytic dependence as well as involvement of other alternative/cumulative agents in such metastatic dissemination. Next, we evaluated invasive potential either upon silencing DRP1 or PDK1, or under 2-DG treated conditions to further verify whether reduction in wound healing in such groups was concomitant with abrogating cell invasion via transwell insert(s). In concordance, DRP1 silencing reduced the invasive potential of these cells (**Figure 4C**, & **Supplementary Figure 3D**), which was also observed in PDK1 silenced groups and also upon 2-DG treatment (**Figures 4D, E** & **Supplementary Figure 3D**).

Keeping in view the importance of epithelial-mesenchymal-transition (EMT) in driving metastasis, chemotherapeutic resistance and cancer-stemness, our next interest was to seek whether disrupting mitochondrial fission hampers EMT at molecular level. To this end we evaluated the effect of either DRP1 or PDK1 silencing, or 2-DG treatment on Epithelial/Neuronal-cadherin (E/N-cadherin) switch, whose dis-regulation is a prominent hallmark of EMT. A significant reduction in N-cadherin as well as vimentin with a concomitant up-regulation in E-cadherin was observed upon DRP1/PDK1 silencing or 2-DG treatment (**Figure 4F**), which indicates a possible role of mitochondrial fission and glycolytic metabolism in inducing EMT in BRAF^V600E^ metastatic CRC (m-CRC). Together these data show glycolytic metabolism as a driver force in inducing metastasis in BRAF^V600E^ CRC, possibly via regulating mitochondrial fission.

## Discussion

**Figure 5:**
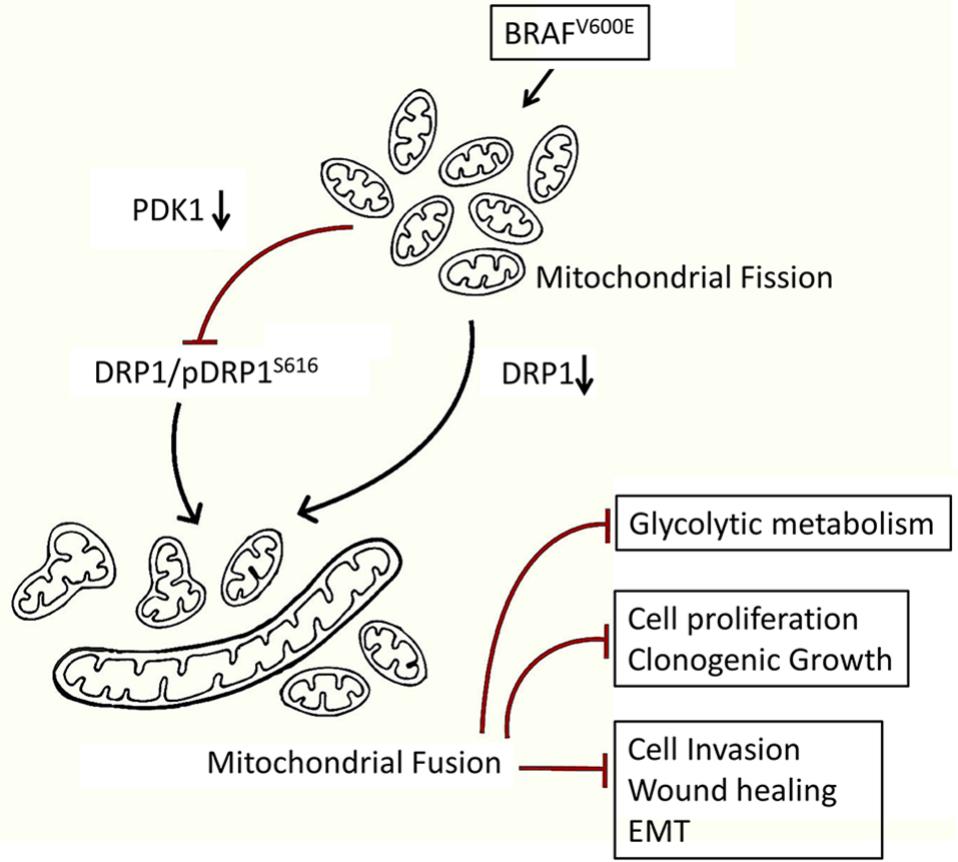
Mechanistic model showing a higher fragmented mitochondrial phenotype in BRAF^V600E^ CRC cells endows a metabolic advantage and tumorigenic propensity to these cells. Inhibition of such fission leads to a reduction in glycolytic metabolism and tumorigenic potential in BRAF^V600E^ CRC cells, highlighting a therapeutic modality towards targeting mitochondrial fission.

Despite a significant progress in treating CRC for past one and a half decade, the disease still represents a chief cause of cancer-related mortality globally (61, 62). The development and inclusion of molecular testing techniques for knowing oncogenic status of RAS, BRAF, and microsatellite instability (MSI) has benefited to better devise treatment regimens, which in turn have improved the clinical outcome (63). Further, it has been known since recently that m-CRC with mutated RAS is resistant to anti-EGFR therapies (64, 65), and those with MSI phenotype are sensitive to immunotherapy (66, 67), but colorectal cancer with BRAF mutation/s is awaiting such a specific chemotherapeutic approach. Though V600E mutation in BRAF gene has been previously reported to confer a fragmented mitochondrial phenotype and drug resistance to melanoma (51, 68), the underlying mechanism/s is not clear, especially with respect to form function relationship. We confirm here that BRAF^V600E^ mutation in endogenous setting also confers a fragmented mitochondrial phenotype to CRC cells over those harboring wild type BRAF (**Figure 1**). The subsequent reduction in mitochondrial mass (increased fusion) led to an inhibition in tumor growth *in vitro* and clonogenic potency in soft agar; suppressed glycolytic and/or anabolic metabolism as well as inhibited metastasis (summarized in **Figure 5**). Here in, our data demonstrate that inhibiting mitochondrial fission takes over the advantage of exploiting such mitochondrial biology driven by BRAF^V600E^ cancers (51, 68).

The data presented here establish a critical role of DRP1/pDRP1^S616^ in regulating mitochondrial function, transformative potency and metastatic dissemination in BRAF^V600E^ CRC. First we demonstrate a direct role of DRP1 in regulating mitochondrial fission in BRAF^V600E^ CRC cells, and provide evidence that such mitochondrial fission confers a growth advantage to these cells. The rescue of mitochondrial fission upon PLX4032 (Vemurafenib) treatment indicates the dependence at least in part, on constitutive MAPK activation downstream of BRAF or KRAS, as observed in some previous studies (34, 48, 50). However, further studies are warranted to decipher the importance of such regulation on the observed effects. Further to support the higher energy needs and maintaining redox state, mitochondrial fission also regulates the higher glucose fermentation rates. Such involvement of DRP1 in tumorigenic potential and metabolic reprograming downstream of KRAS/BRAF is supported by some previous studies in separate models of PDAC and melanoma (48, 50). However, the former study implicated it via suppressing glycolytic metabolism while the latter observed it via reducing OXPHOS. Our results however show that BRAF^V600E^ CRC cells are more glycolytic and undergo extensive mitochondrial fission to support their anabolic needs, a characteristic of most solid cancers (69, 70). Such observations are an indication of a dual metabolic regulation either presumably dependent on cellular environment (the cells being exposed to) or the cell type. Further, a recent study demonstrated the therapeutic relevance of targeting mitochondrial fission factor DRP1 in BRAF^V600E^ melanoma (51); however, it didn’t show any metabolic correlation despite a more glycolytic nature of BRAF^V600E^ cancers (71). The present study however demonstrates a pro-tumorigenic role of DRP1 in BRAF^V600E^ CRC, and shows that BRAF^V600E^ dichotomizes from BRAF^WT^ CRC cells both in tumorigenicity as well as metabolic preference (**Figure 2** & **Supplementary Figure 1**), thus adding that such dichotomy could be because of difference in metabolism (72). Such an inhibition in tumor growth upon inhibiting mitochondrial fission has recently been reported in some cancers (48, 68), and the results are in agreement with other previous studies as well (11, 34, 50, 51).

We recognize that PDK1 is a glycolytic gatekeeper which maintains the desired glycolytic phenotype of cancerous cells by obstructing the conversion of pyruvate (generated in glycolysis) to acetyl coenzyme A (Acetyl CoA) (43), and impeding oxidation of pyruvate to generate cellular energy. Further, as a key glycolytic marker PDK1 is reported to favor cell proliferation, metastases and worst prognosis (73–75), thereby making it an important therapeutic target in cancer. In line our results demonstrate that PDK1 could be an important glycolytic regulator in BRAF^V600E^ driven CRC and that it also could pose a therapeutic vulnerability to cancers driven via fragmented mitochondrial phenotype (**Figures 3** & **Supplementary Figure 2**). While knocking down PDK1 abrogated cellular proliferation/clonogenicity, it did not show much reduction in cellular migration/invasion compared to DRP1 knockdown, a probable indication of interplay of multiple compensatory pathways regulating mitochondrial fission/fusion dynamics other than PDK1 (37, 50). Further the antagonistic effects of PDK1 silencing on cell proliferation/clonogenicity could be attributed to reduction in glucose fermentation rates, otherwise necessary to ensure continued generation of ATP and molecular building blocks of proliferating cells (69, 70). The results seem in corroboration with previously reported role of PDK1 in maintaining cell proliferation and antagonizing apoptosis (76), though we couldn’t evaluate apoptosis in the present study. More studies are needed to decipher whether and how mitochondrial fission influences the apoptosis antagonizing nature of PDK1 in both BRAF^V600E^ CRC and other cancers.

Cancer metastasis, a multistep and indeed a multifactorial process accounts for nearly 90% of all cancer related deaths worldwide (77). EMT (epithelial mesenchymal transition) marks the early steps during metastasis characterized by formation of circulating tumor cells (CTCs) in blood, linked to worst prognosis in various cancers (78–80). About 20% of newly diagnosed CRC cases are metastatic and another 20% will develop into metastatic (m-CRC), giving rise to significant reduction in survival rates (81, 82). In the present study we found a significant reduction in cellular invasion upon DRP1 inhibition, with a concomitant elevation in epithelial marker E-cadherin and down-regulation of mesenchymal markers viz Vimentin and N-cadherin. These results were further confirmed by a reduction in wound healing capacity concomitant with the restoration of epithelial cellular morphology, post DRP1 silencing. Besides, we also observed a correlation between mitochondrial fission mediated metabolic reprogramming and metastasis/EMT. The evidence that some key metabolic enzymes including PDK1 regulate cancer metastasis (73, 83), and that the mitochondria being the central regulators of cellular glucose metabolism, we speculate that such metabolic shift must be coordinated with the organelle function in accordance with mitochondrial dynamics. In our study, we demonstrated that DRP1 inhibition tempered the metastatic potential of BRAF^V600E^ CRC cells by modulating glucose fermentation rate, which could be ascribed to mitochondrial fusion. These results were further supported by using either glycolytic inhibitor 2-DG or silencing PDK1 which could significantly modulate EMT switch as well as cellular invasion/migration. There are previous reports which connect mitochondrial fission to breast cancer metastasis however, a metabolic explanation was lacking (58). Here we add that such increased mitochondrial fission could be attributed to maintain higher glycolytic rate for ensuring surplus ATP production to meet increased energy demands of migrating cells (73). Overall, our study reveals that DRP1 inhibition modulated BRAF^V600E^ mediated CRC progression by reduction in mitochondrial fission, which in-turn modulated the glycolytic metabolism necessary for such tumorigenesis.

## Data Availability Statement

The original contributions presented in the study are included in the article/**Supplementary Material**. Further inquiries can be directed to the corresponding author.

## Author Contributions

RP and MH conceptualized the idea and designed the experiments. RP and ZB. performed most of the experiments and acquired the data with assistance from ZA who helped in the cell proliferation, transfections, and maintenance. RP performed confocal imaging and data acquisition with assistance from NS. RP wrote the manuscript under supervision of MH with inputs from ZB. ZA sketched the mechanistic interpretation under guidance from RP. MH provided all the infrastructure and reagents to conduct the research. All authors contributed to the article and approved the submitted version.

## Funding

The research received no external funding support; however, some support was utilized from other extra mural funding grant of MH (Ministry of AYUSH, Government of India grant no. 3-7/2009-CCRUM/EMR). The Indian Council of Medical Research (ICMR) is highly acknowledged for providing fellowship (ICMR- SRF; 3/2/2/356/2016/NCDIII) to RP.

## Conflict of Interest

The authors declare that the research was conducted in the absence of any commercial or financial relationships that could be construed as a potential conflict of interest.
