# Supplementary material for: DRP1 Promotes BRAFV600E-Driven Tumor Progression and Metabolic Reprogramming in Colorectal Cancer
Source: Front Oncol. 2021 Mar 2;10:592130. doi: 10.3389/fonc.2020.592130 (PMC7961078; doi:10.3389/fonc.2020.592130)
Supplement: Supplementary file 1 [file DataSheet_1.pdf]

Supplementary File:

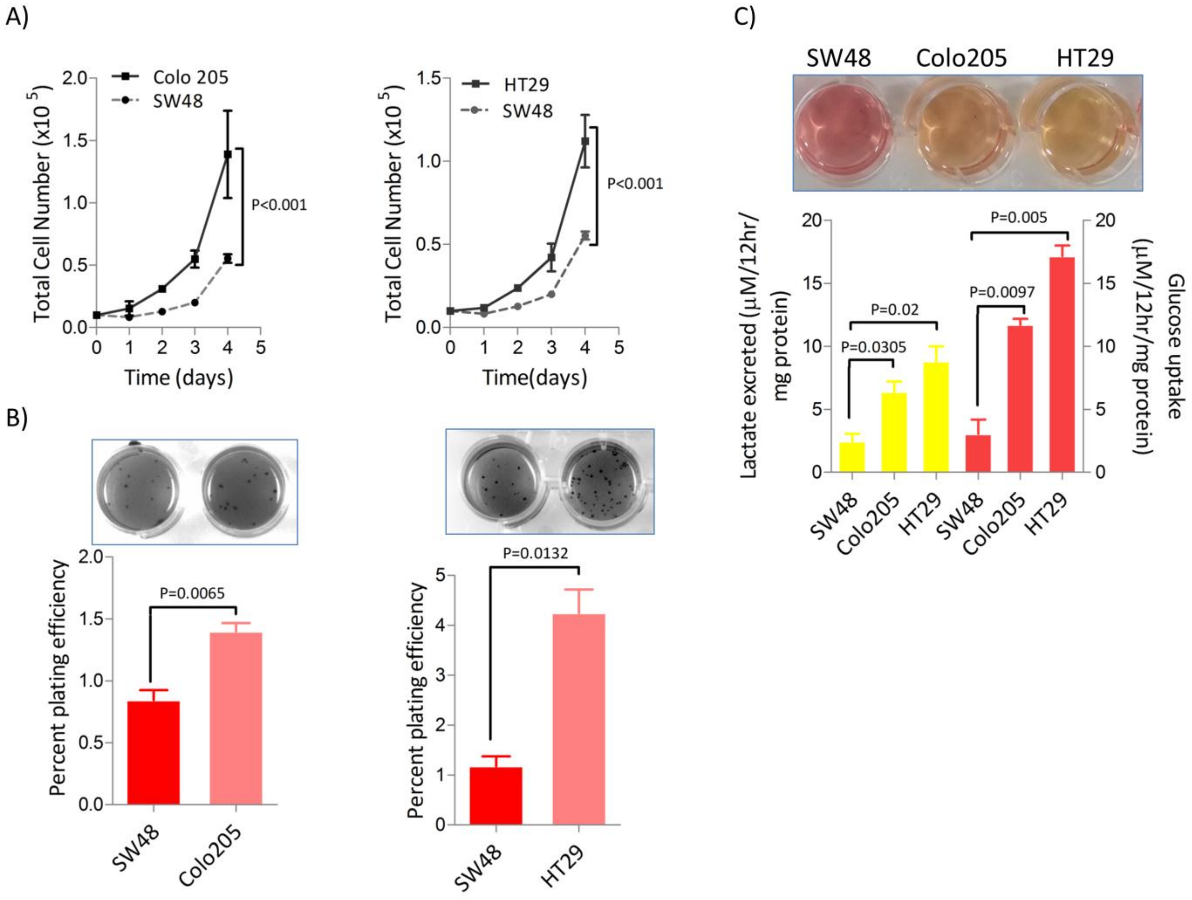

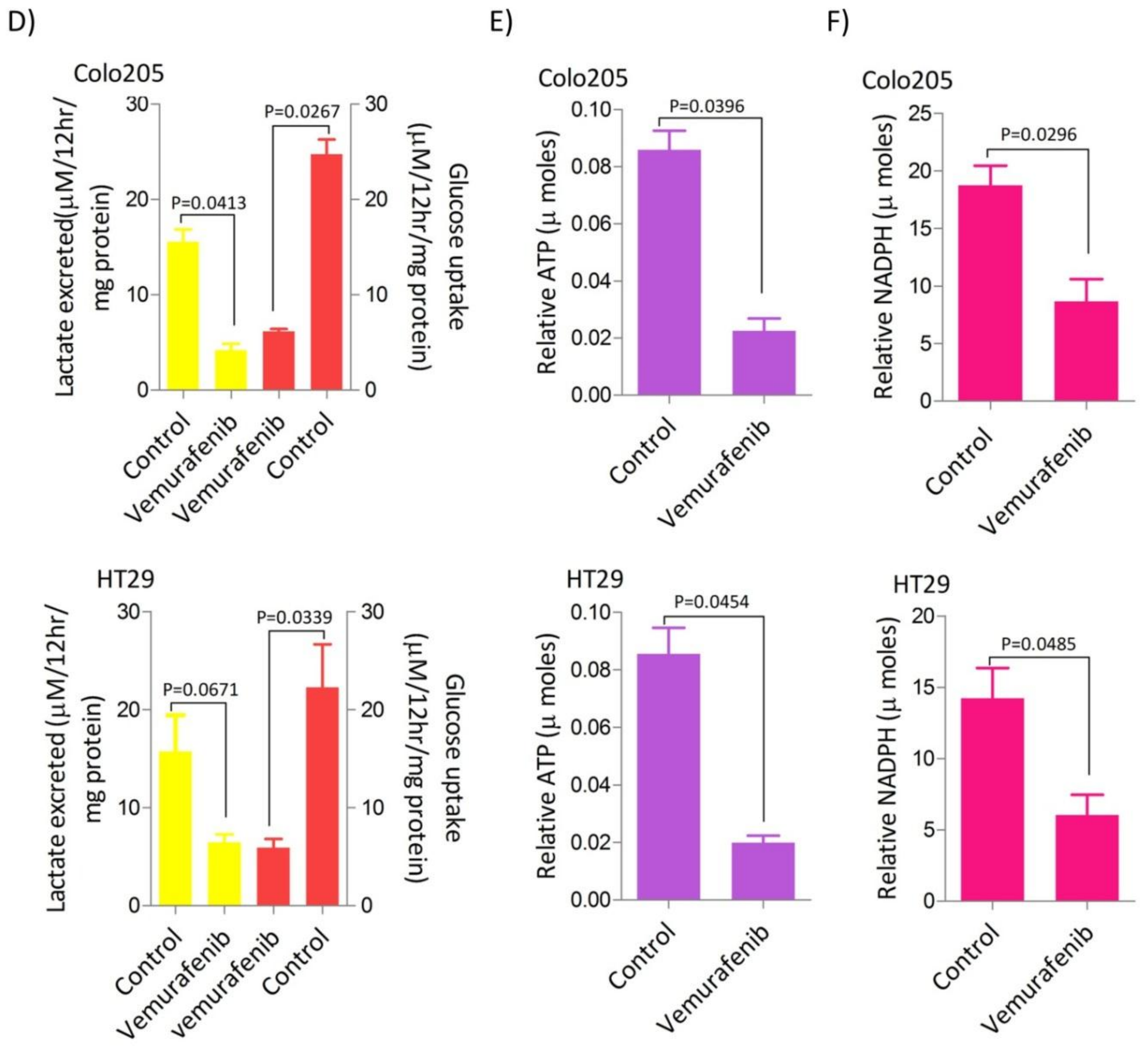

Supplementary Figure 1: More fragmented mitochondrial phenotype leads to a more tumorigenic potential and reprogramming in glucose metabolism in $BRAF^{V600E}$ CRC cells.

A) $BRAF^{V600E}$ CRC cells have higher proliferation rate/s compared to $BRAF^{WT}$ type (n=4, mean± SEM), statistical analysis was done by 2 way ANOVA, followed by Bonferroni post-tests comparing replicate means by row with corresponding p values shown. **B)** Higher clonogenic growth in soft agar by $BRAF^{V600E}$ compared to $BRAF^{WT}$ CRC cells (n=3, mean± SEM), statistical analysis was done by unpaired t-test followed by Welch's correction assuming unequal variance in means, corresponding p values shown. **C)** Higher lactate production and glucose utilization rate(s) by $BRAF^{V600E}$ than $BRAF^{WT}$ cells (evidenced by a more yellowish soft agar by Colo 205 and HT 29 compared to SW 48 cells), (n=3, mean± SEM), statistical analysis was done by unpaired t-test, corresponding p values shown. Reduction in **D)** glucose uptake and lactate production, **E)** ATP generation, and **F)** NADPH production post vemurafenib treatment in $BRAF^{V600E}$ cells (n=3, mean± SEM), statistical analysis was done by unpaired t-test, followed by Welch's correction assuming unequal variance in means, corresponding p values shown.

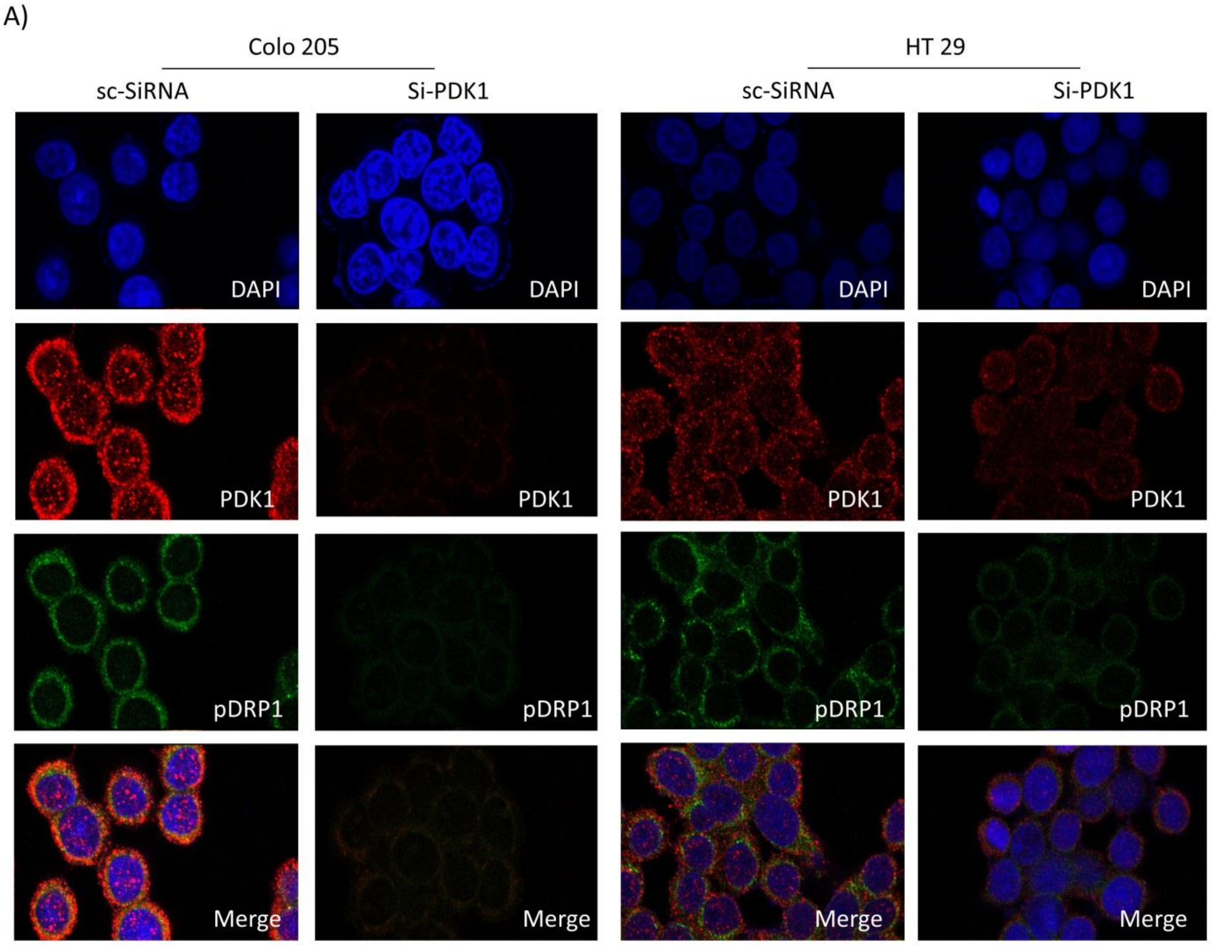
A)
Colo 205
HT 29
sc-SiRNA
Si-PDK1
sc-SiRNA
Si-PDK1
DAPI
PDK1
pDRP1
Merge

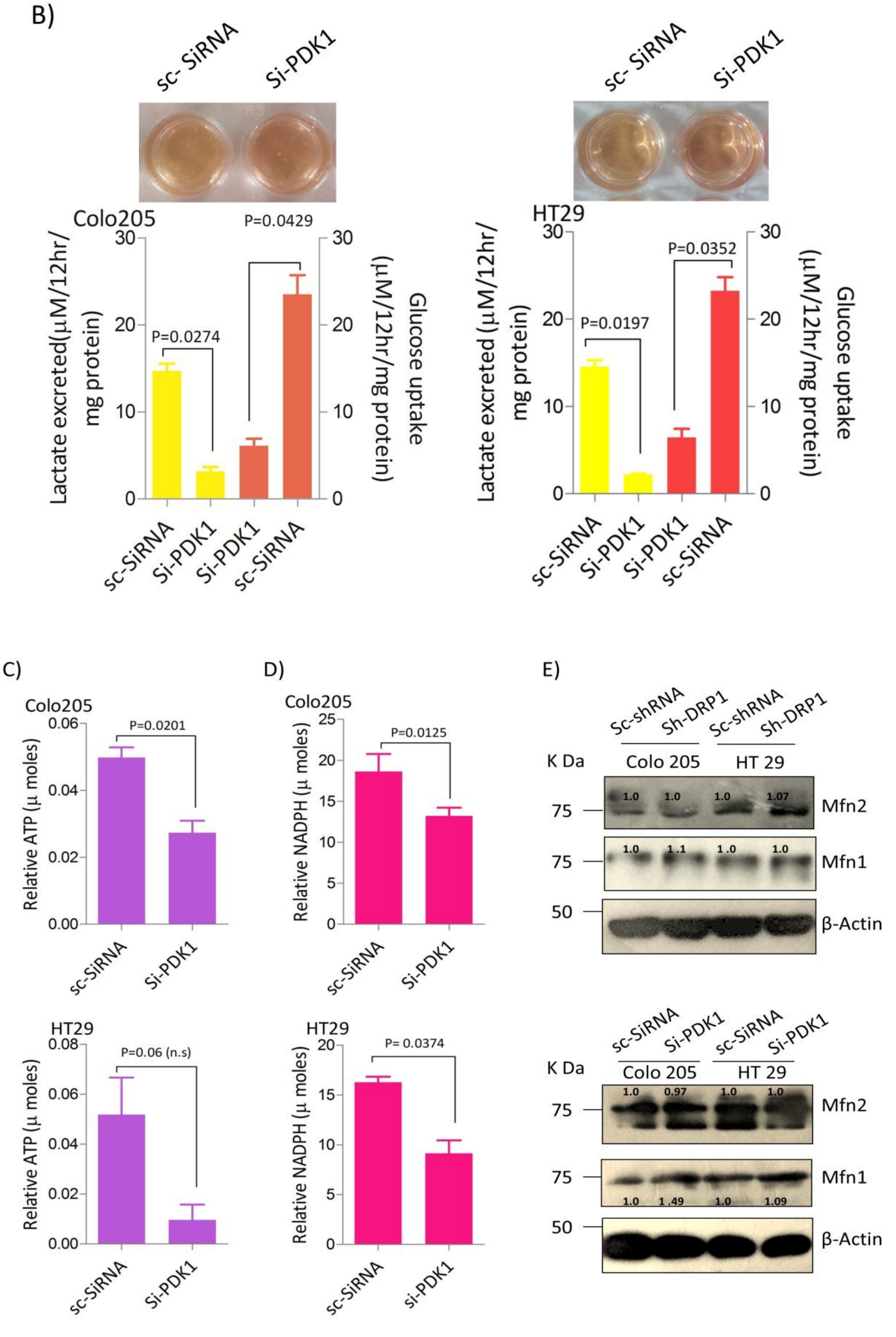

Supplementary Figure 2: Pyruvate dehydrogenase kinase 1 (PDK 1) regulates DRP1 mediated mitochondrial fission and glycolytic phenotype in BRAF^{V600E} cells.

A) Representative confocal Immunofluorescent images (2.5x zoom, 60x original magnification) of $BRAF^{V600E}$ CRC cells post PDK1 silencing showing a reduction in $pDRP1^{S616}$ staining on PDK1 silencing, red represents PDK1 staining while as green represents $pDRP1^{S616}$ staining and blue represents DAPI (nuclei) (n=3). **B)** Reduction in lactate production and Glucose consumption rates post PDK1 silencing in $BRAF^{V600E}$ CRC cells (n=3, mean± SEM), supported by a decrease in yellowing of soft agar post PDK1 silencing. **C, D)** Relative ATP and NADPH levels post PDK1 silencing in $BRAF^{V600E}$ CRC cells (n=3, mean± SEM); statistical analysis was done by unpaired t-test followed by Welch's correction assuming unequal variance in means, corresponding p values shown. **E)** Western blots showing no effect of silencing PDK1 or DRP1 on fusion mediators Mfn 1 & 2.

A)

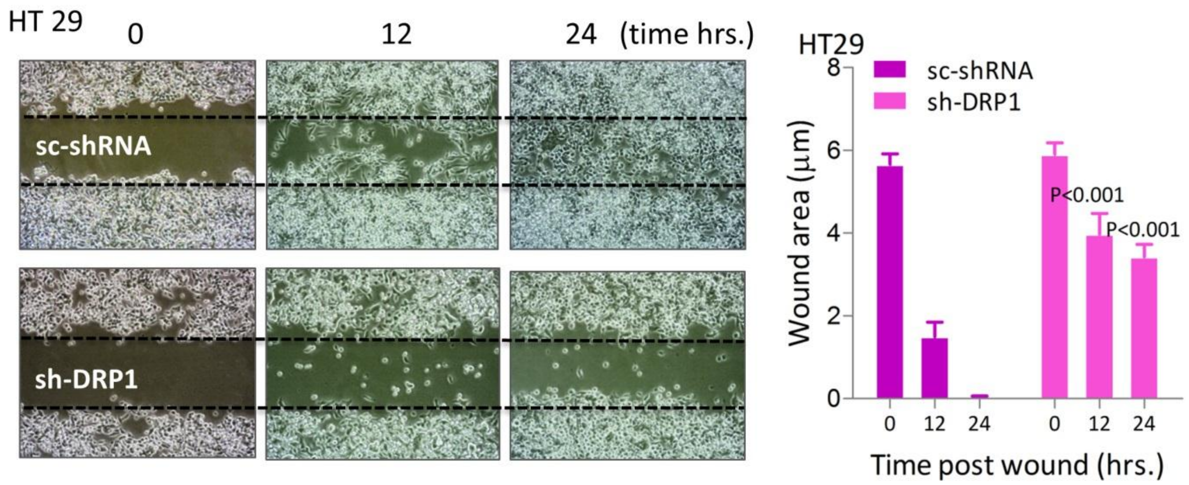

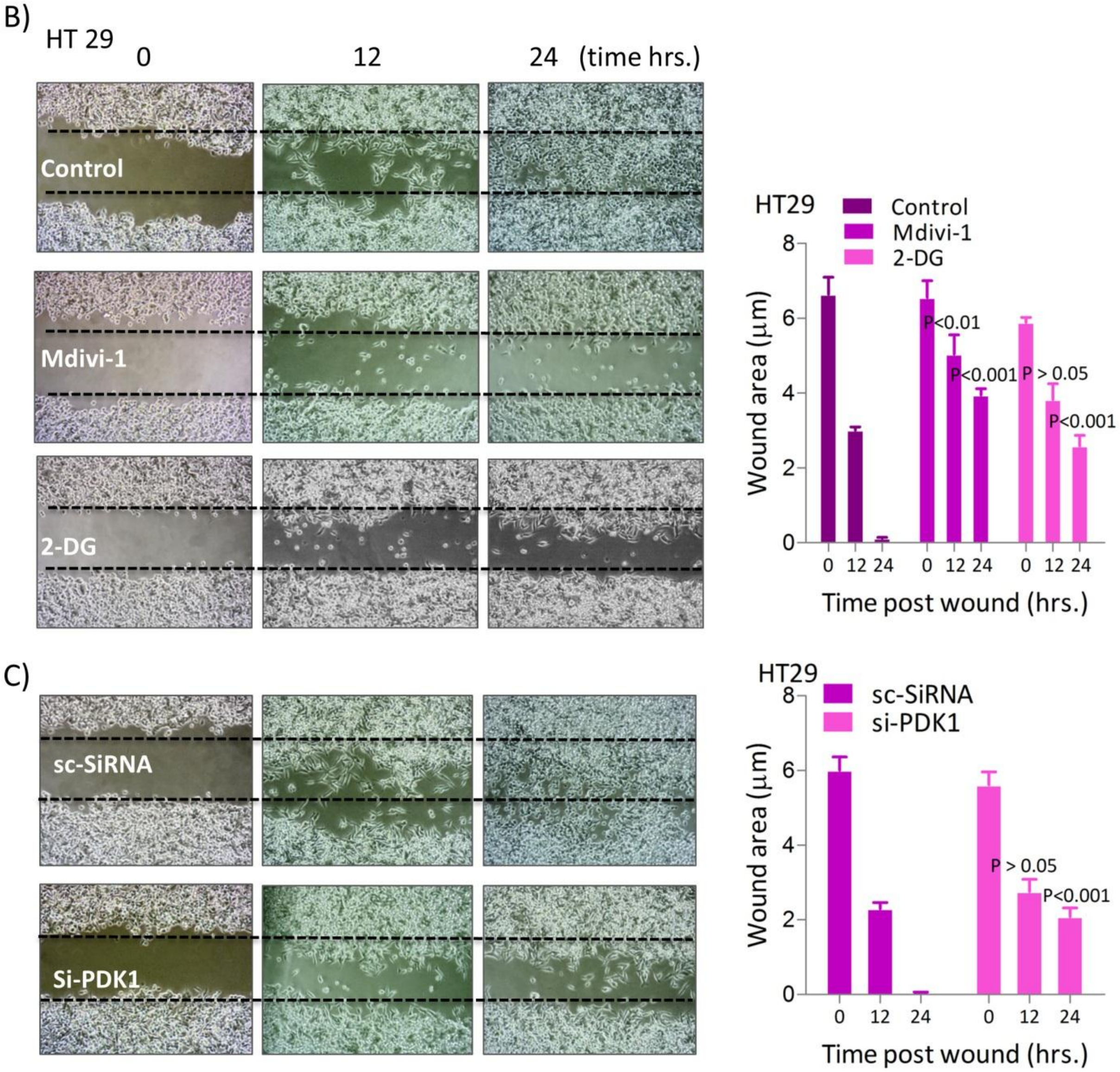
B)
HT 29
0
12
24 (time hrs.)
Control
Mdivi-1
2-DG
HT29
Control
Mdivi-1
2-DG
Wound area (μm)
P<0.01
P<0.001
P > 0.05
P<0.001
Time post wound (hrs.)
C)
sc-SiRNA
Si-PDK1
HT29
sc-SiRNA
si-PDK1
Wound area (μm)
P > 0.05
P<0.001
Time post wound (hrs.)

D)

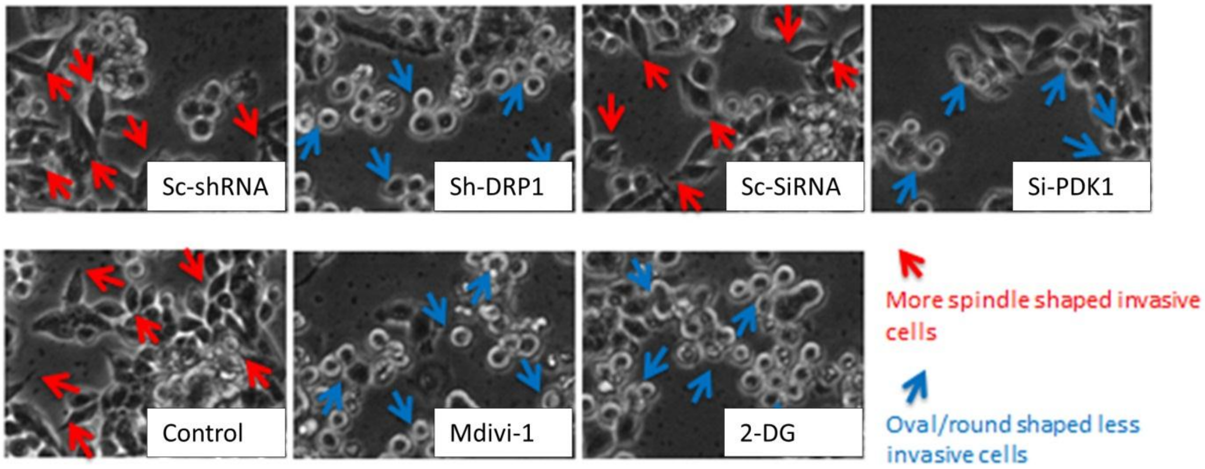

Supplementary Figure 3: Mitochondrial fission regulates migration and invasion in $BRAF^{V600E}$ CRC cells through glucose metabolic reprograming.

Relative cell migration/wound healing rates at indicated time points post- **A)** DRP1 silencing, **B)** 2-deoxyglucose treatment, and **C)** PDK1 silencing in HT29 cells, (n=3, mean± SEM); statistical analysis was done by 2-way ANOVA followed by Bonferroni post-tests comparing replicate means by row with corresponding p values shown. **D)** Changes in cellular morphologies showing an induction of epithelial (round/oval) contrary to mesenchymal (spindle) shape in the indicated groups.

Uncropped western blots:

A)

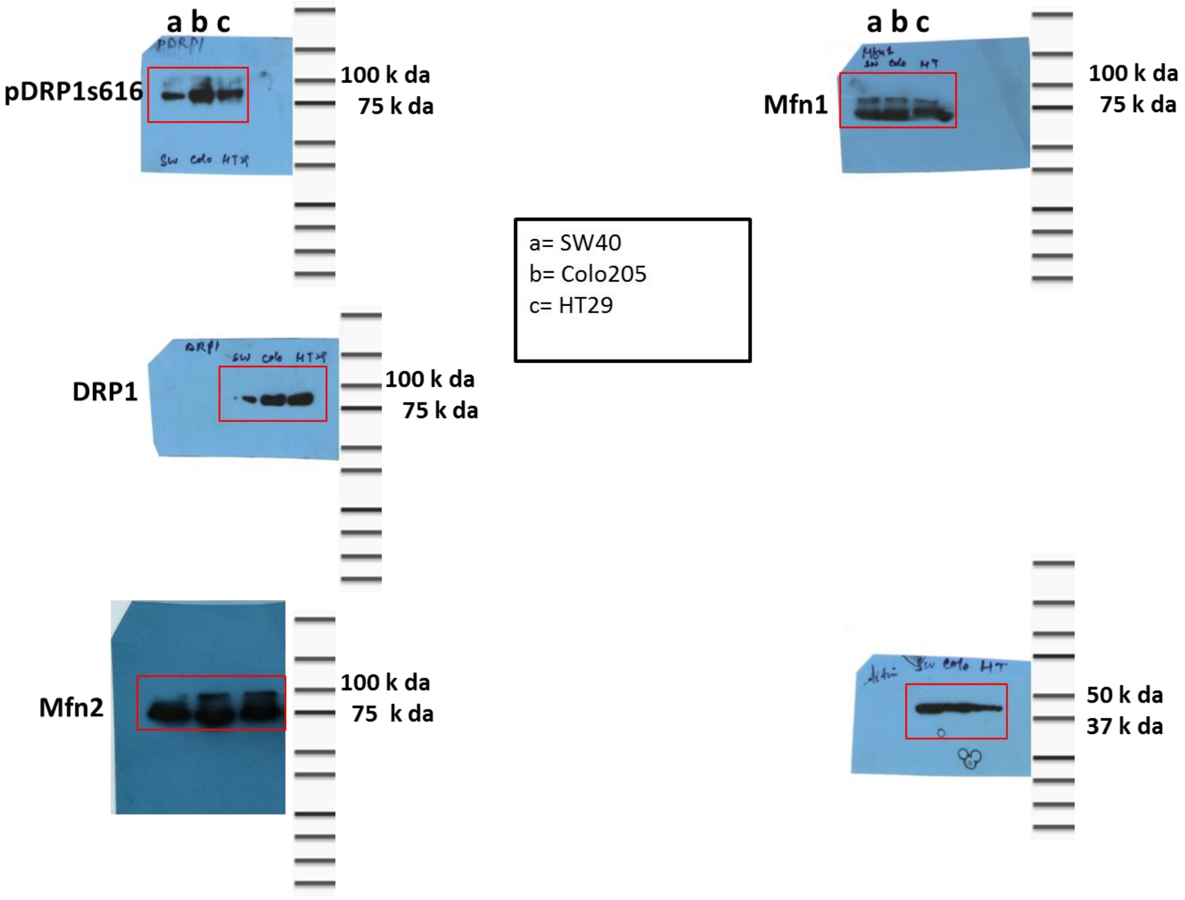

B)

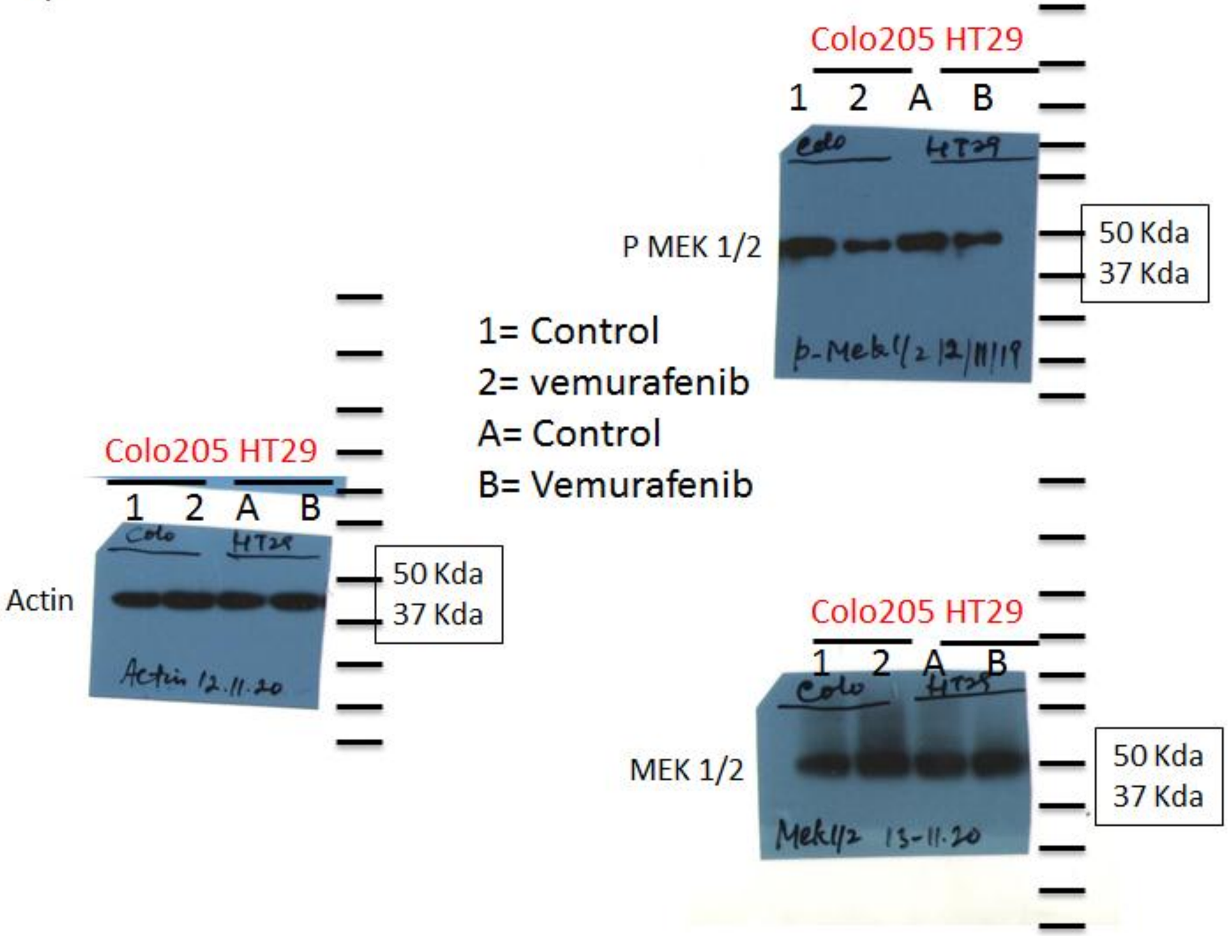

Colo205 HT29
1 2 A B
P MEK 1/2
50 Kda
37 Kda
1= Control
2= vemurafenib
A= Control
B= Vemurafenib
Colo205 HT29
1 2 A B
Actin
50 Kda
37 Kda
Colo205 HT29
1 2 A B
MEK 1/2
50 Kda
37 Kda

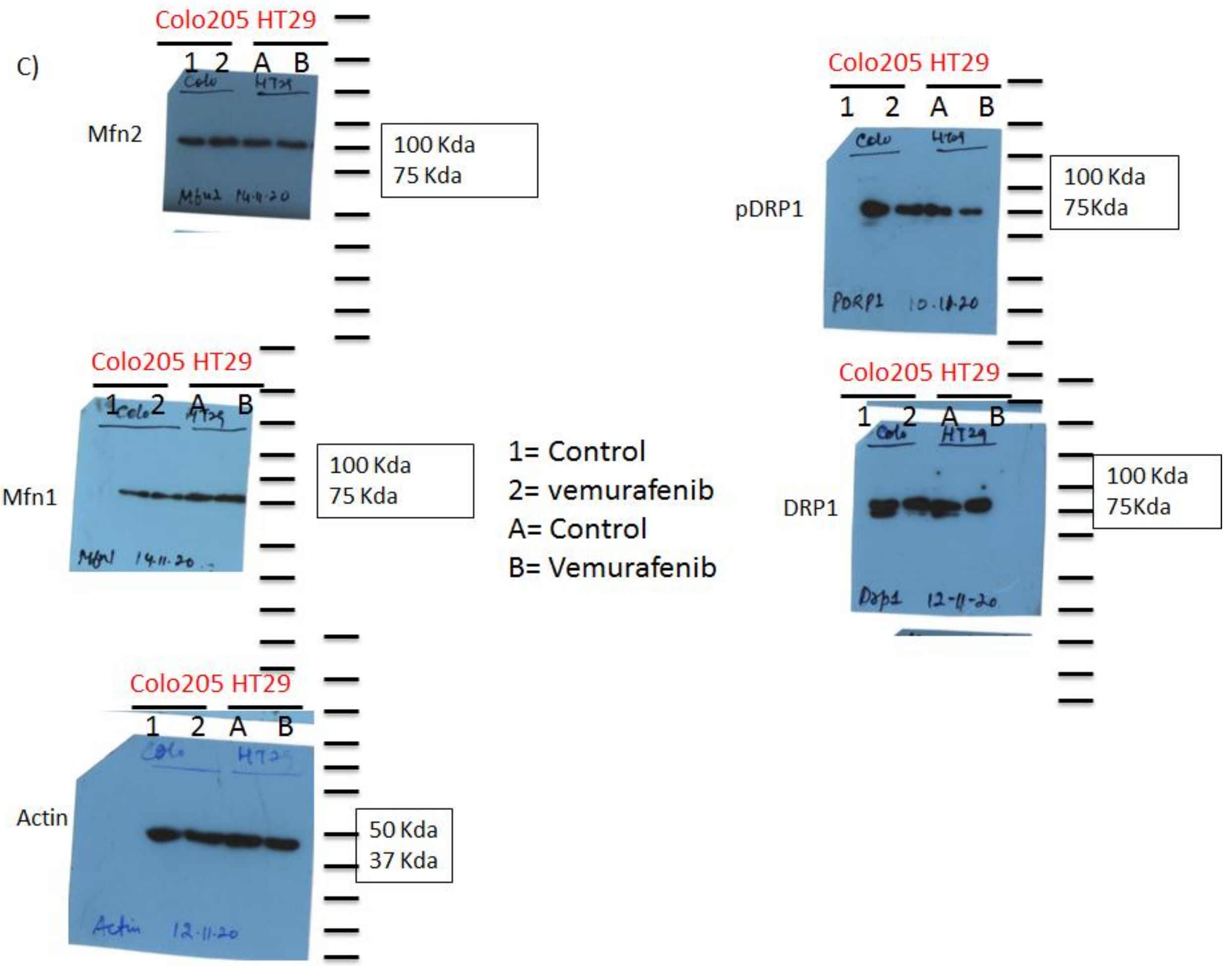
C)
Colo205 HT29
1 2 A B
Mfn2
100 Kda
75 Kda
Mfn1
100 Kda
75 Kda
Actin
50 Kda
37 Kda
1= Control
2= vemurafenib
A= Control
B= Vemurafenib
pDRP1
100 Kda
75Kda
DRP1
100 Kda
75Kda

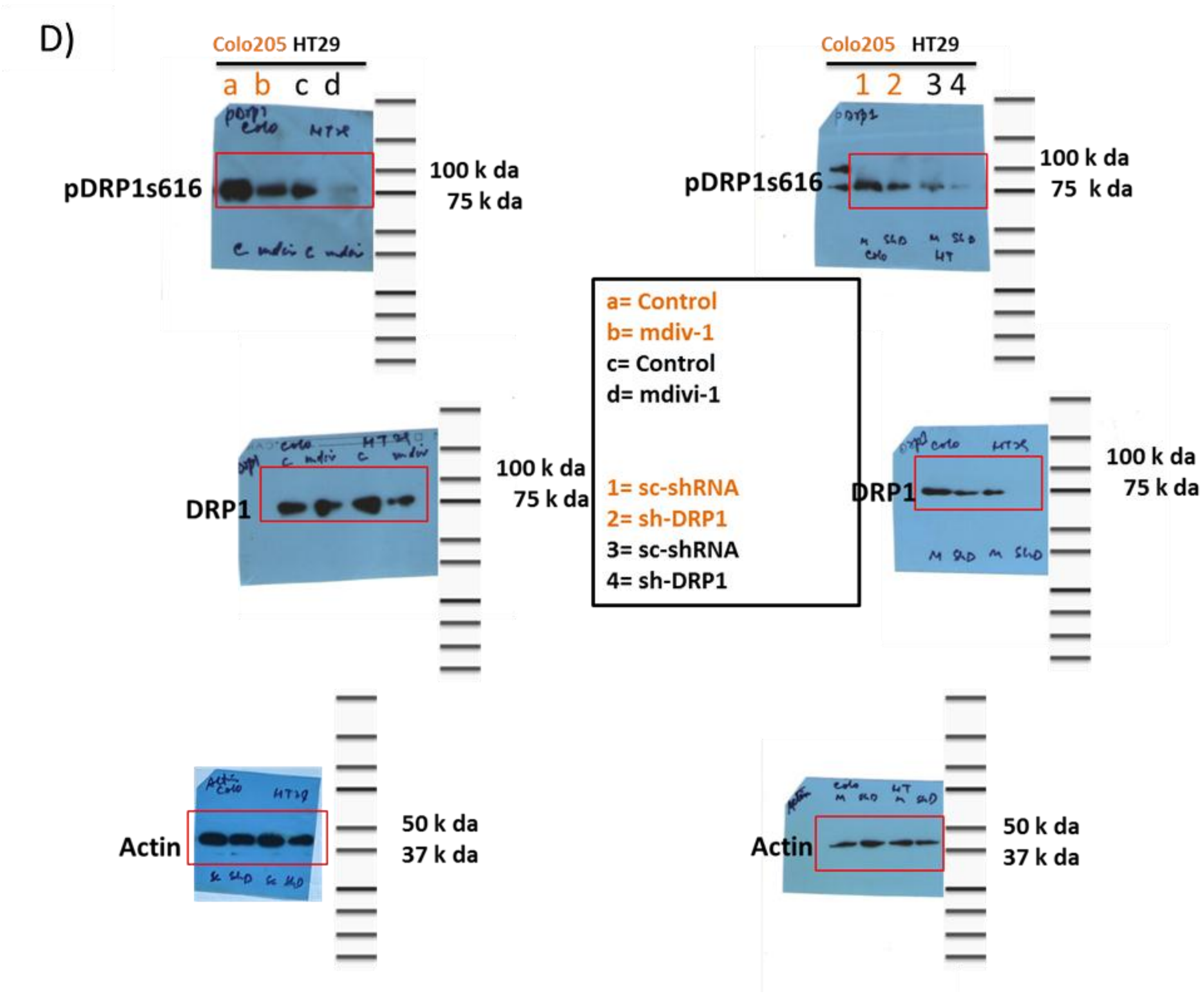

D)
Colo205 HT29
a b c d
pDRP1s616
100 k da
75 k da
DRP1
100 k da
75 k da
Actin
50 k da
37 k da
Colo205 HT29
1 2 3 4
pDRP1s616
100 k da
75 k da
DRP1
100 k da
75 k da
Actin
50 k da
37 k da
a= Control
b= mdiv-1
c= Control
d= mdivi-1
1= sc-shRNA
2= sh-DRP1
3= sc-shRNA
4= sh-DRP1

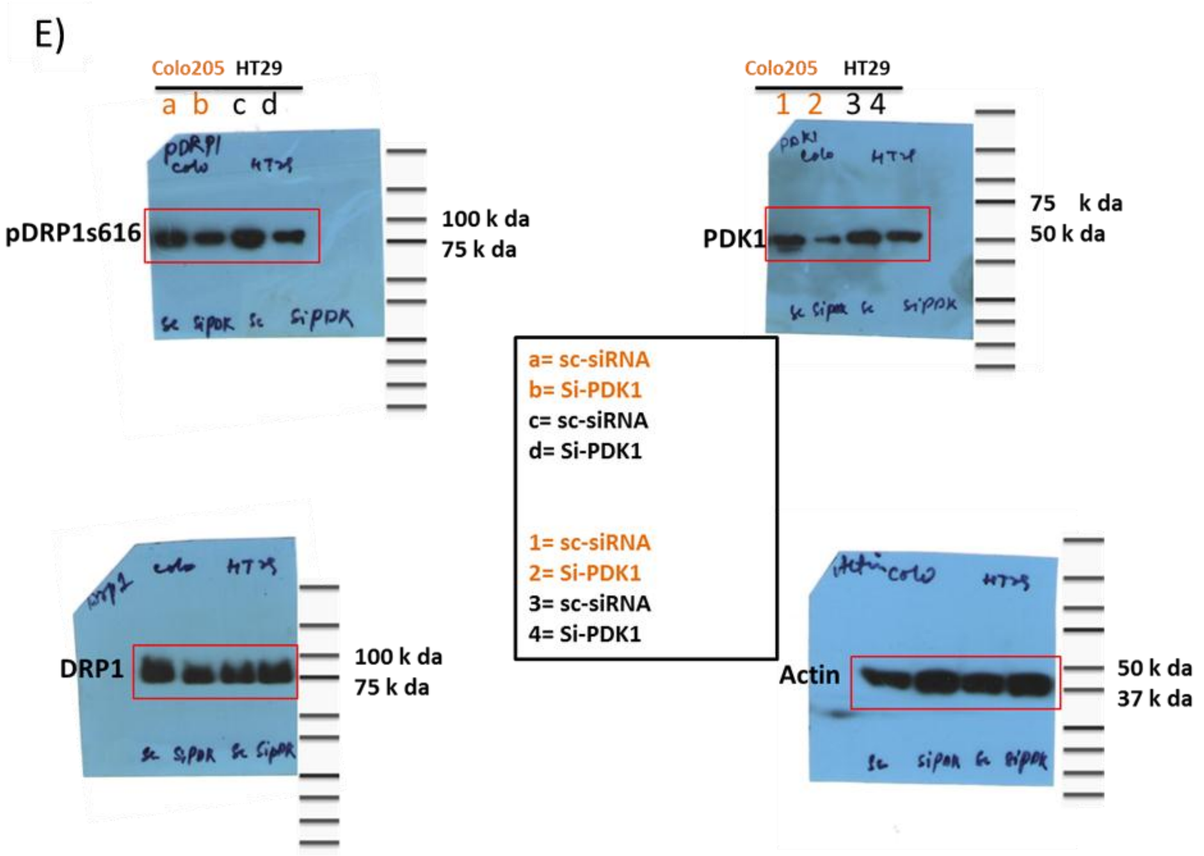
E)
Colo205 HT29
a b c d
pDRP1s616
100 k da
75 k da
Colo205 HT29
1 2 3 4
PDK1
75 k da
50 k da
a= sc-siRNA
b= Si-PDK1
c= sc-siRNA
d= Si-PDK1
1= sc-siRNA
2= Si-PDK1
3= sc-siRNA
4= Si-PDK1
DRP1
100 k da
75 k da
Actin
50 k da
37 k da

F)

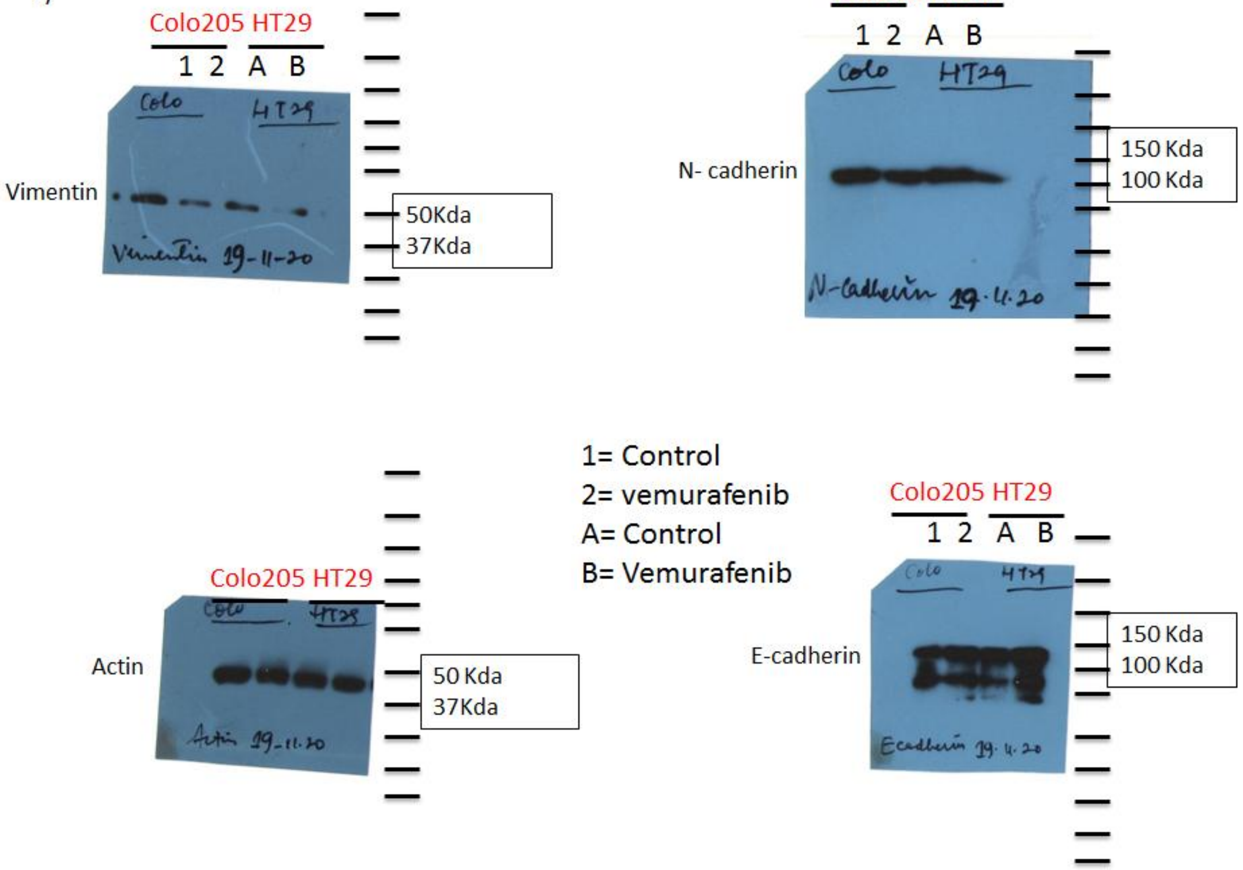

Colo205 HT29
1 2 A B
Vimentin
50Kda
37Kda
Colo205 HT29
1 2 A B
N- cadherin
150 Kda
100 Kda
Colo205 HT29
Actin
50 Kda
37Kda
1= Control
2= vemurafenib
A= Control
B= Vemurafenib
Colo205 HT29
1 2 A B
E-cadherin
150 Kda
100 Kda

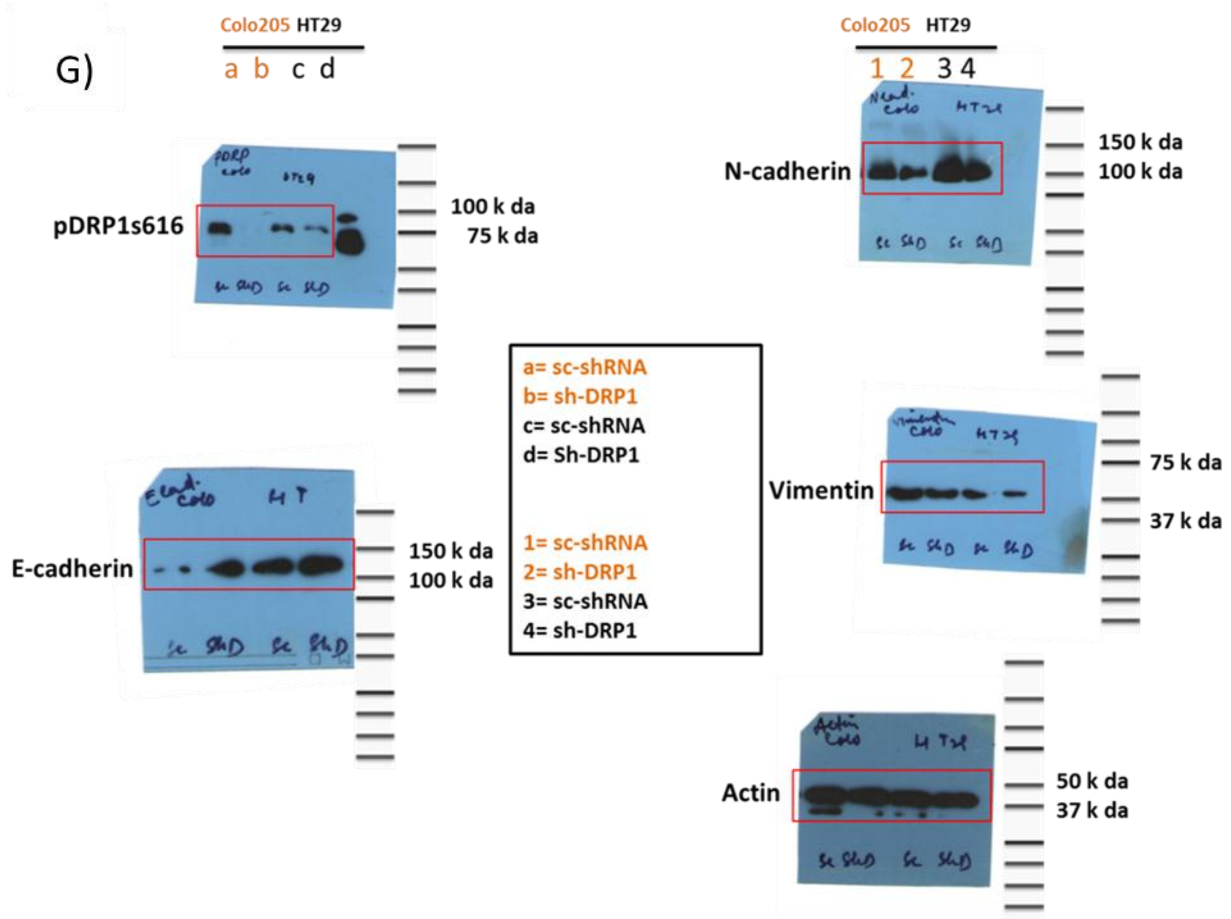
G)
Colo205 HT29
a b c d
pDRP1s616
100 k da
75 k da
E-cadherin
150 k da
100 k da
a= sc-shRNA
b= sh-DRP1
c= sc-shRNA
d= Sh-DRP1
1= sc-shRNA
2= sh-DRP1
3= sc-shRNA
4= sh-DRP1
Colo205 HT29
1 2 3 4
N-cadherin
150 k da
100 k da
Vimentin
75 k da
37 k da
Actin
50 k da
37 k da

H)

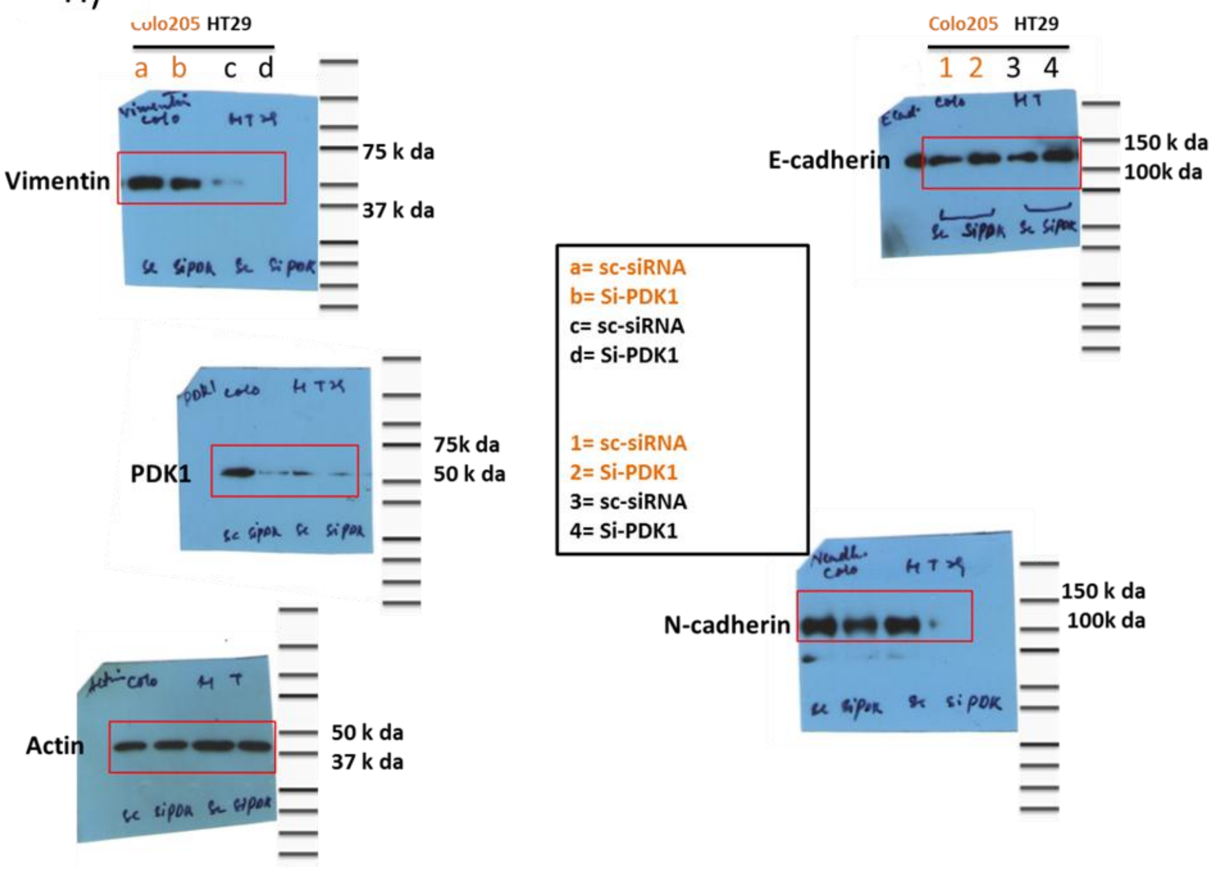
Colo205 HT29
a b c d
Vimentin
75 k da
37 k da
PDK1
75k da
50 k da
Actin
50 k da
37 k da
a= sc-siRNA
b= Si-PDK1
c= sc-siRNA
d= Si-PDK1
1= sc-siRNA
2= Si-PDK1
3= sc-siRNA
4= Si-PDK1
Colo205 HT29
1 2 3 4
E-cadherin
150 k da
100k da
N-cadherin
150 k da
100k da

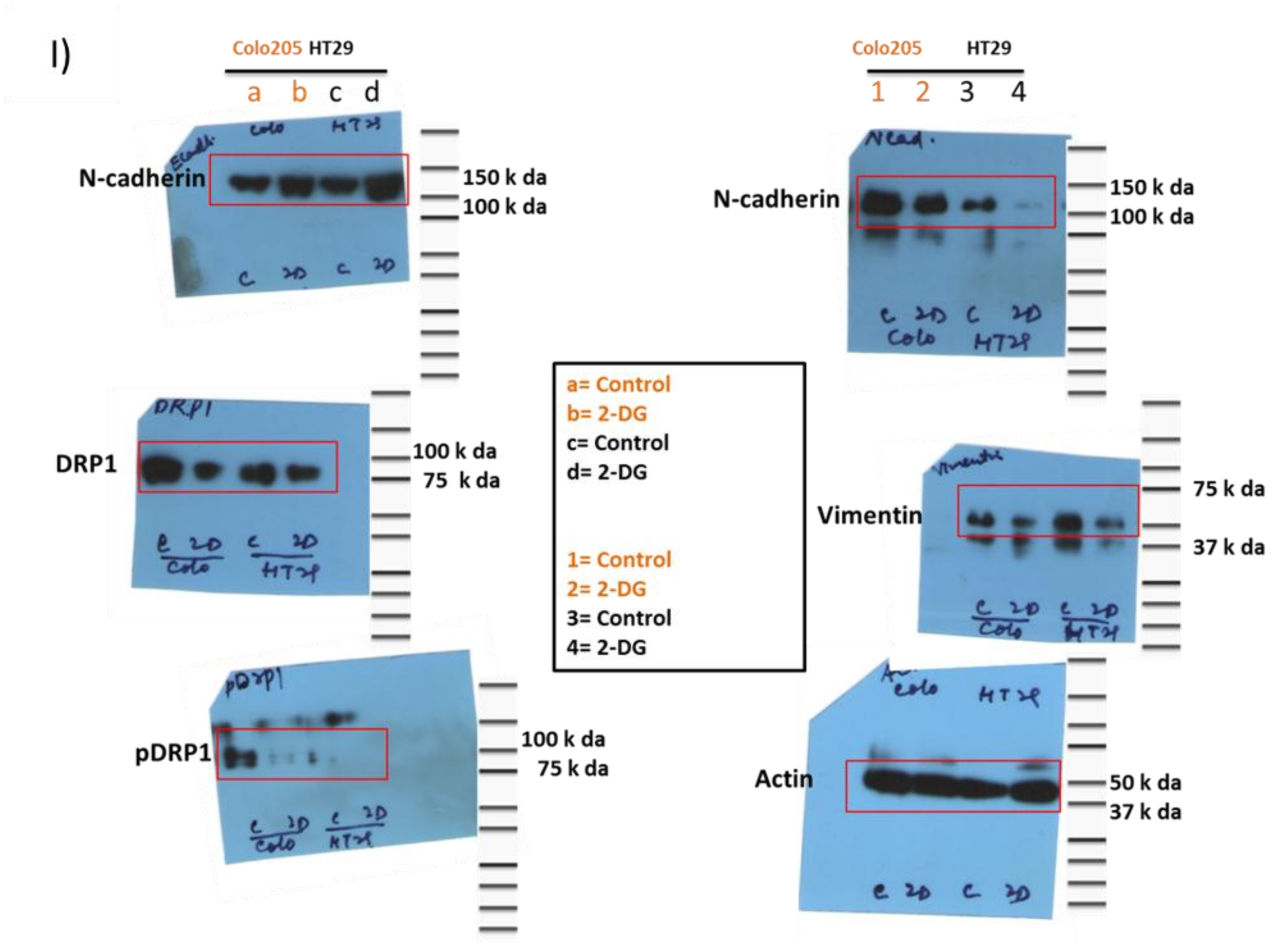
I)
Colo205 HT29
a b c d
N-cadherin
150 k da
100 k da
DRP1
100 k da
75 k da
pDRP1
100 k da
75 k da
a= Control
b= 2-DG
c= Control
d= 2-DG
1= Control
2= 2-DG
3= Control
4= 2-DG
Colo205
HT29
1 2 3 4
N-cadherin
150 k da
100 k da
Vimentin
75 k da
37 k da
Actin
50 k da
37 k da

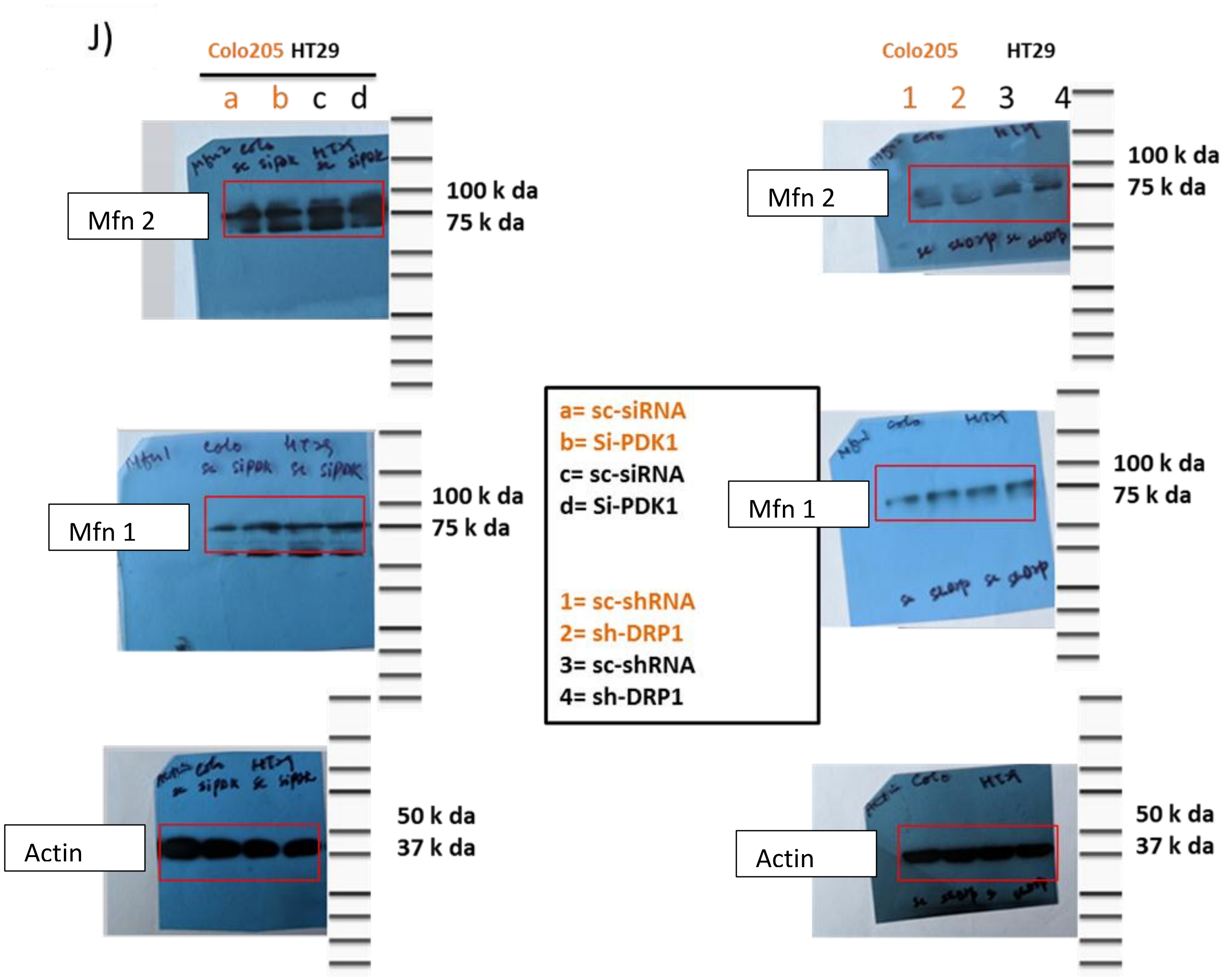

Supplementary Figure 4: A-J) Representative uncropped western blot images corresponding to those shown in main text as **Figure 1B (S4A); 1C(S4B); 1D(S4C); 2C (S4D); 3A (S4E); 4B (S4F); 4F(SG-I)** and **S2E (S4J)** respectively.

Note: In the uncropped images, we have copy and pasted the picture of the Protein ladder (Precision plus dual color from Biorad) owing to the fact that x-ray film seldom/very least develops the ladder bands on it. The highlighted bands in red colored rectangles represent the approximate widths of the membrane where the latter was cut with a scissor after blocking to probe multiple strips from a single membrane as possible.
